# Thermally Gated Dual‐Cascade Nanozyme for Enhanced Mild‐Temperature Photothermal Therapy

**DOI:** 10.1002/advs.202517528

**Published:** 2025-11-07

**Authors:** Shuyu Wang, Shenghui Wang, Mengyuan Cao, Lulu Wang, Wei Jiang, Xiyun Yan, Ying Liu, Bing Jiang

**Affiliations:** ^1^ Nanozyme Laboratory in Zhongyuan, School of Basic Medical Sciences Zhengzhou University Zhengzhou 450001 China; ^2^ National Health Commission Cardiovascular Disease Regenerative Medicine Research Key Laboratory Central China Subcenter of National Center for Cardiovascular Diseases Henan Cardiovascular Disease Center, Fuwai Central‐China Cardiovascular Hospital Central China Fuwai Hospital of Zhengzhou University Zhengzhou 450046 China; ^3^ Nanozyme Laboratory in Zhongyuan Henan Academy of Innovations in Medical Science Zhengzhou Henan 451163 China; ^4^ CAS Engineering Laboratory for Nanozyme, Key Laboratory of Biomacromolecules, Institute of Biophysics Chinese Academy of Sciences Beijing 100101 China

**Keywords:** cascade catalytic nanozyme, heat shock protein 70, mild‐temperature photothermal therapy, thermo‐responsive behavior, tumor catalytic therapy

## Abstract

Mild‐temperature photothermal therapy (mPTT) is attractive for cancer treatment due to its safety and precision but is limited by heat shock protein 70 (HSP70)‐mediated thermotolerance, which allows tumor cells to survive sublethal heating. Herein, a thermo‐responsive cascade nanozyme system is reported (Ru‐GOx‐PNN) that enhances mPTT through dual suppression of HSP70. This system integrates a Ru‐doped porous carbon framework that serves as both an efficient NIR photothermal transducer and a catalytic nanozyme with intrinsic peroxidase (POD)‐ and catalase (CAT)‐like activities, immobilized glucose oxidase (GOx) to initiate metabolic oxidation and supply H_2_O_2_, and an outer thermosensitive PNN hydrogel that gates substrate access and exposes active sites specifically within the mPTT window. Upon mild heating, the GOx‐POD cascade generates hydroxyl radicals that trigger lipid peroxidation and destabilize HSP70, while the GOx‐CAT cascade consumes glucose and reduces adenosine triphosphate synthesis, further suppressing HSP70 expression. The resulting inhibition of HSP70 relieves its restraint on c‐Jun N‐terminal kinase signaling, thereby amplifying apoptosis. Collectively, this strategy effectively overcomes thermal resistance and enhances the therapeutic efficacy of mPTT. This study highlights the potential of cascade catalytic nanozymes as intelligent platforms for safe, efficient, and precise tumor therapy, particularly in challenging malignancies such as esophageal squamous cell carcinoma (ESCC).

## Graphical abstract

A thermo‐responsive cascade nanozyme (Ru‐GOx‐PNN) synergistically enhances mild photothermal therapy by dual suppression of HSP70 through ROS‐induced lipid peroxidation and ATP depletion. This intelligent system effectively overcomes thermotolerance and amplifies apoptosis, offering a safe and precise strategy for tumor treatment.

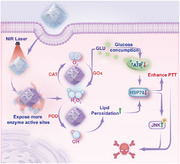

## 1 Introduction

Mild‐temperature photothermal therapy (mPTT; 42–45 °C) offers a favorable safety profile by minimizing collateral thermal injury while enabling localized, light‐guided intervention.^[^ ^1^, ^2^, ^3^, ^4^, ^5^ ^]^ Nevertheless, its clinical promise has not translated into durable tumor control because sublethal hyperthermia rapidly induces thermotolerance.^[^ ^6^, ^7^, ^8^ ^]^ Central to this response is heat shock proteins (HSPs), especially heat shock protein 7 (HSP70), which plays a pivotal role in mPTT resistance.^[^ ^9^ ^]^ Under mild hyperthermia, HSP70 helps stabilize mitochondrial integrity, maintain proteostasis, and suppress apoptosis, thereby directly counteracting mPTT‐induced cytotoxicity.^[^ ^10^, ^11^, ^12^ ^]^ Moreover, HSP70 regulates the folding and activity of multiple proteins, including oncogenic clients, thereby supporting tumor cell survival.^[^ ^13^, ^14^, ^15^ ^]^

A direct path to enhancing mPTT is therefore to disable this HSP70 “thermal shield.” However, existing countermeasures have not fully resolved this impasse. Small‐molecule HSP70 inhibitors (such as glycyrrhizic acid, quercetin, etc.) and siRNA‐based approaches have been explored to sensitize tumors to mPTT by disrupting the ATPase cycle of HSP70, inhibiting its activity, or silencing its gene expression.^[^ ^8^, ^9^, ^16^ ^]^ Nevertheless, small‐molecule inhibitors face significant challenges, including the strong adenosine triphosphate (ATP) / adenoosine diphosphate (ADP) affinity of HSP70, the presence of multiple highly conserved isoforms, and its ubiquitous expression in normal tissues, all of which lead to poor selectivity, systemic toxicity, and incomplete inhibition.^[^ ^15^ ^]^ In contrast, siRNA‐based strategies offer higher specificity but suffer from instability during systemic delivery and long‐term biosafety concerns.^[^ ^9^ ^]^ These limitations underscore the urgent need for alternative, tumor‐confined, and on‐demand strategies to modulate HSP70.^[^ ^17^ ^]^ For example, our Ru‐GOx‐PNN nanozyme system can effectively suppress HSP70 under mild photothermal conditions. Although elevating PTT temperatures above ∼50 °C can restore cytotoxicity but sacrifices selectivity through thermal diffusion and tissue damage.^[^ ^18^, ^19^, ^20^ ^]^ Accordingly, there is a pressing need for a tumor‐confined, on‐demand strategy that weakens HSP70‐mediated thermotolerance without abandoning the mild‐temperature window. This need is especially acute in indications that are amenable to localized light delivery.

Esophageal squamous cell carcinoma (ESCC) exemplifies such a setting. The esophagus is endoscopically accessible, enabling localized near‐infrared (NIR) delivery and real‐time thermal monitoring‐features that naturally favor spatially precise mPTT.^[^ ^21^, ^22^ ^]^ At the same time, ESCC is a highly aggressive malignancy, and conventional treatments such as surgery, chemotherapy, radiotherapy, and immunotherapy continue to yield limited outcomes.^[^ ^23^, ^24^ ^]^ These clinical challenges underscore the need for more effective and safer therapeutic options.^[^ ^25^ ^]^ If HSP70‐mediated thermotolerance can be dismantled under mild hyperthermia, mPTT could provide a gentle yet effective option for ESCC. Realizing this promise requires a mechanism that suppresses HSP70 within the mPTT window in a tumor‐confined manner.

Mechanistic evidence supports a dual‐pathway approach to suppress HSP70 under mild hyperthermia. First, reactive oxygen species (ROS), particularly hydroxyl radicals (•OH), play pivotal roles in inducing lipid peroxidation (LPO), which promotes HSP70 degradation either via direct interaction or by initiating intracellular oxidative stress pathways.^[^ ^26^, ^27^, ^28^, ^29^, ^30^, ^31^ ^]^ Second, HSP70 expression and function are tightly regulated by intracellular ATP levels.^[^ ^32^, ^33^ ^]^ Depletion of ATP markedly suppresses HSP70 activity, weakening cellular defenses against thermal and oxidative stress.^[^ ^34^ ^]^ Glucose oxidase (GOx) offers a metabolic approach to downregulate HSP70 by consuming glucose and inhibiting glycolytic ATP production.^[^ ^35^ ^]^ Together, these mechanisms motivate a dual‐targeting strategy to suppress HSP70 through simultaneous promotion of its degradation via ROS‐induced LPO and metabolic inhibition of its expression via ATP depletion, thereby enhancing mPTT efficacy. Moreover, previous studies have shown that HSP70, as a molecular chaperone, can modulate cellular stress responses by interacting with the c ‐ Jun N ‐ terminal kinase (JNK) pathway, thereby inhibiting excessive activation of stress signaling.^[^ ^36^ ^]^ JNK serves as a critical intracellular stress‐response mediator that regulates apoptosis, proliferation, and inflammation.^[^ ^37^ ^]^ Under normal conditions, HSP70 restrains JNK activation to maintain homeostasis; however, when HSP70 is downregulated or functionally impaired, JNK signaling is unleashed, leading to activation of downstream pro‐apoptotic pathways. In this context, nanozymes, particularly cascade catalytic nanozymes, provide an attractive platform to integrate multiple enzyme‐like functions within a single nanostructure, thereby enabling synergistic regulation of oxidative and metabolic pathways.^[^ ^38^, ^39^, ^40^ ^]^

Building on this rationale, we present a mild photothermal‐triggered, temperature‐responsive nanozyme system (Ru‐GOx‐PNN) for ESCC. A temperature‐sensitive polymer (PNN) acts as a programmable thermal “switch,” exposing catalytic sites only within the 42–45 °C window. Upon NIR‐induced mild heating, two coordinated cascades are activated, a GOx‐peroxidase (GOx‐POD) branch that converts hydrogen peroxide (H_2_O_2_) into •OH to trigger LPO‐driven HSP70 degradation, and a GOx‐catalase (GOx‐CAT) branch that recycles oxygen (O_2_) and depletes glucose to suppress ATP production, thereby downregulating HSP70 expression. Beyond direct HSP70 suppression, the nanozyme also modulates downstream signaling pathways, HSP70 downregulation relieves inhibition of the JNK pathway, promoting JNK phosphorylation and apoptosis induction. This synergistic interplay between physical mild photothermal treatment and biological regulation substantially enhances therapeutic efficacy. Because activation is restricted to the heated region, spatial selectivity is preserved while thermotolerance is dismantled.

**Scheme 1:**
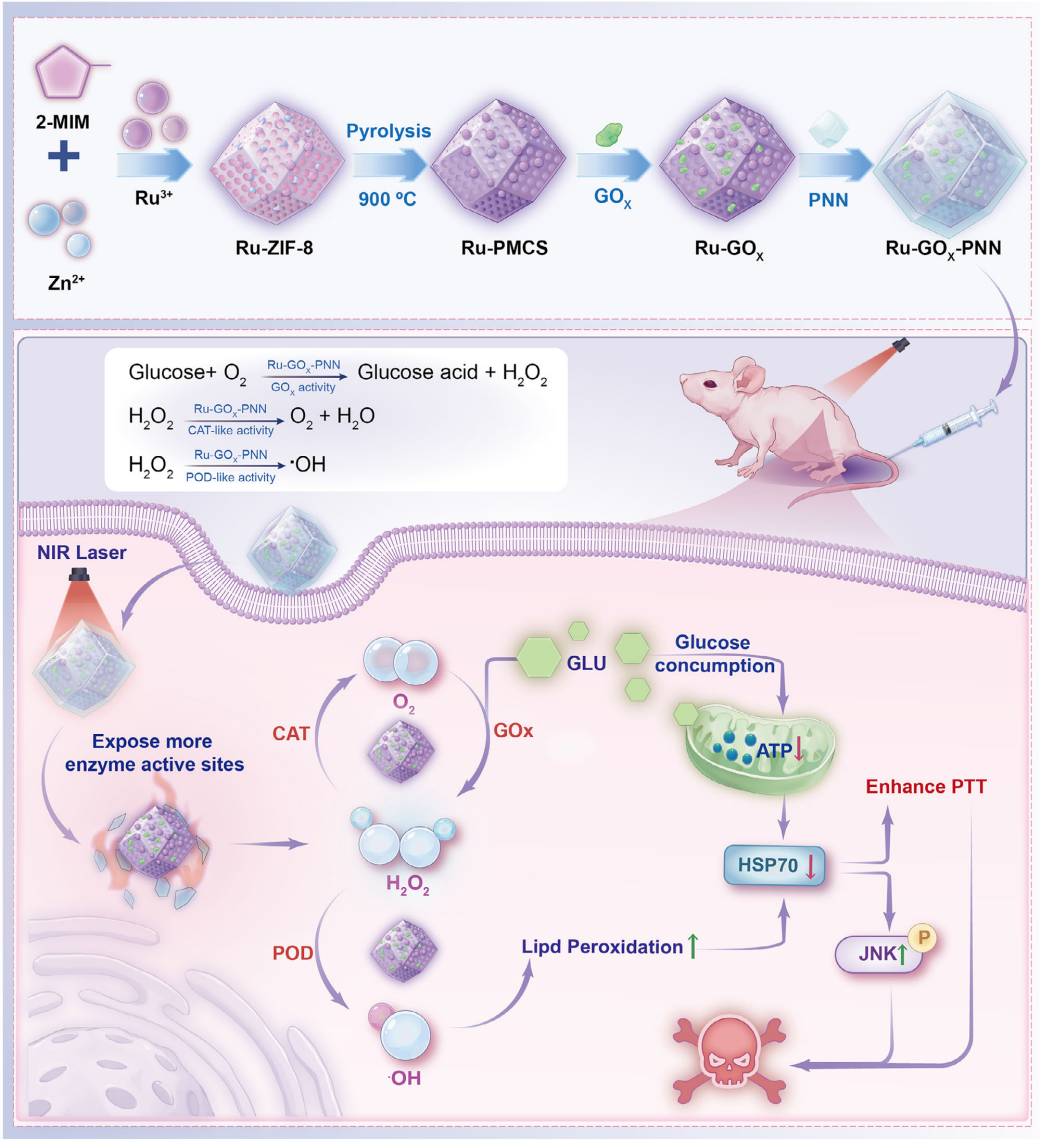
Schematic illustration of the temperature‐responsive Ru‐GOx‐PNN nanozyme system for enhancing mPTT. Under mild hyperthermia triggered by NIR irradiation, the phase‐transition polymeric network (PNN) exposes the catalytic sites of Ru and GOx, activating the cascade catalytic reactions. The GOx‐POD cascade generates •OH, inducing lipid peroxidation and promoting HSP70 degradation, while the GOx‐CAT cascade depletes glucose and inhibits ATP synthesis, further downregulating HSP70 expression. The dual suppression of HSP70 alleviates thermotolerance and activates the JNK signaling pathway, resulting in enhanced tumor cell apoptosis and improved therapeutic efficacy of mPTT.

In summary, the temperature‐responsive Ru‐GOx‐PNN nanozyme system achieves marked improvement in mPTT efficacy through three key advantages, (i) photothermal‐responsive catalytic activation via temperature‐controlled exposure of enzyme active sites, enhancing catalytic efficiency; (ii) cascade amplification through multi‐enzyme activities inducing lipid peroxidation and ATP depletion, resulting in dual suppression of HSP70; and (iii) enhanced tumor cell apoptosis through HSP70 inhibition coupled with phosphorylated JNK (p‐JNK) activation (**Scheme** **1**). These advances position Ru‐GOx‐PNN as a rational platform for selective, effective mPTT in ESCC and potentially other solid tumors.

## 2 Results and Discussion

### 2.1 Structural Characterization and Photothermal Performance of Ru‐GOx‐PNN Nanozymes

**Figure 1:**
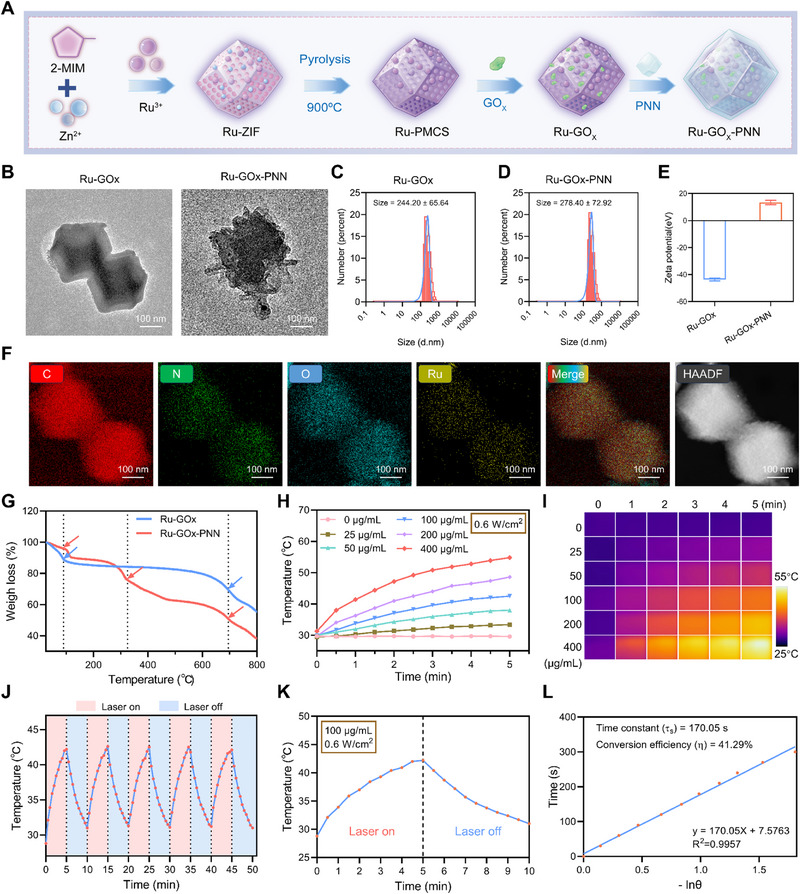
Structural Characterization and Photothermal Properties of Ru‐GOx‐PNN Nanozymes. A) Schematic illustration of the synthetic route for Ru‐GOx‐PNN nanozymes. B) TEM images of Ru‐GOx and Ru‐GOx‐PNN nanozymes. Scale bar = 100 nm. C–E) Hydrodynamic diameter and zeta potential of Ru‐GOx and Ru‐GOx‐PNN nanozymes determined by DLS. F) EDS element diagram of Ru‐GOx‐PNN nanozymes. Scale = 100 nm. G) Thermogram analysis curves of Ru‐GOx and Ru‐GOX‐PNN nanozymes. H) Temperature curves and I) corresponding infrared thermal images of Ru‐GOx‐PNN nanozyme at varying concentrations under NIR irradiation (808 nm, 0.6 W·cm^−2^ for 5 min). J) Evaluation of the photothermal stability of Ru‐GOx‐PNN nanozymes at multiple on/off laser cycles (808 nm, 0.6 W cm^−2^). K) Temperature curves of Ru‐GOx‐PNN nanozymes at a single on/off laser cycle (808 nm, 0.6 W cm^−2^). L) The photothermal conversion efficiency (η) of Ru‐GOx‐PNN nanozymes calculated by linear fitting based on the cooling data during the laser off period.

To dismantle HSP70‐mediated thermotolerance under mild hyperthermia, we designed a temperature‐responsive cascade nanozyme (Ru‐GOx‐PNN) that integrates a Ru‐doped porous carbon framework derived from ZIF‐8, GOx, and a thermosensitive poly (N‐isopropylacrylamide‐co‐N‐(hydroxymethyl)acrylamide) (PNN) hydrogel coating (**Figure** **1**A). The Ru‐doped porous carbon not only provides structural robustness and high surface area but also exhibits intrinsic peroxidase (POD)‐ and catalase (CAT)‐like activities together with efficient NIR photothermal conversion. Incorporation of GOx within the mesoporous architecture further enables metabolic oxidation and H_2_O_2_ generation to fuel cascade reactions, while the outer PNN hydrogel shell endows temperature‐gated exposure of catalytic sites, restricting catalytic activation to the mild hyperthermia window of 42–45 °C. To enable direct comparison, a control nanozyme Ru‐GOx without the PNN coating was also synthesized. This modular strategy was conceived to couple efficient NIR‐to‐heat conversion with dual enzymatic cascades (GOx‐POD and GOx‐CAT) under spatiotemporal control.

Transmission electron microscopy (TEM) showed that both Ru‐GOx and Ru‐GOx‐PNN retained the dodecahedral morphology characteristic of ZIF‐8, indicating structural integrity during synthesis (Figure 1B). Dynamic light scattering (DLS) measurements showed an increase in hydrodynamic diameter from 244.20 ± 65.64 nm (Ru‐GOx) to 278.40 ± 72.92 nm (Ru‐GOx‐PNN) after PNN coating (Figure 1C,D). Concurrently, zeta potential reversed from ‐43.58 ± 0.98 mV to +13.37 ± 1.63 mV (Figure 1E), attributed to protonation of tertiary amine groups from the TEMED initiator in the PNN network. Elemental mapping (EDS) confirmed homogeneous C, N, O, and Ru distribution (Figure 1F), and X ‐ ray photoelectron spectroscopy (XPS) validated component incorporation (Figure S1, Supporting Information).Notably, the high‐resolution Ru 3d XPS spectrum revealed a distinct Ru 3d_5/2_ peak characteristic of Ru^2+^ (Figure S2, Supporting Information), indicating partial reduction of Ru^3+^ to Ru^2+^ under the pyrolytic conditions, consistent with the coordination environment stabilized by the ZIF‐8 framework. X‐ray diffraction (XRD) patterns displayed broad peaks at 25° and 44°, with no significant change after GOx loading or PNN coating, confirming framework stability (Figure S3, Supporting Information).

The thermal stability and chemical composition of Ru‐GOx and Ru‐GOx‐PNN were further assessed via thermogravimetric analysis (TGA) and Fourier‐transform infrared (FTIR) spectroscopy. TGA profiles revealed a two‐step mass loss for Ru‐GOx, whereas Ru‐GOx‐PNN exhibited an additional degradation step, consistent with the presence of the PNN hydrogel layer (Figure 1G). FT‐IR spectra of Ru‐GOx and Ru‐GOx‐PNN (Figure S4, Supporting Information) confirm PNN coating on Ru‐GOx. For Ru‐GOx, peaks at 3417 cm^−1^ (O‐H), 2973/2923 cm^−1^ (C‐H, ‐CH_2_‐), and 1627 cm^−1^ (amide II N‐H/C‐N) verify GOx presence. Ru‐GOx‐PNN shows 3388 cm^−1^ (O‐H), 2925/2856/2771 cm^−1^ (C‐H), 1731 cm^−1^ (C = O, from PNN amide), 1151 cm^−1^ (C‐O, from ‐CH_2_OH), and 673/630 cm^−1^ (C‐H/N‐H bending), indicating PNN incorporation. Red‐shifted/broadened O‐H, weakened 1627 cm^−1^ peak, and increased/split C‐H peaks suggest N‐H···O ═ C hydrogen bonds between GOx amides and PNN C ═ O, plus diverse alkyl environments from PNN. These confirm robust hydrogen‐bonded PNN loading on Ru‐GOx, ensuring structural stabilization and integration.

Differential scanning calorimetry (DSC) indicated that Ru‐GOx‐PNN exhibits a phase transition temperature of 44.61 °C with an enthalpy change of 52.04 J g^−1^, lower than that of pure PNN (55.25 °C, 24.61 J g^−1^), reflecting enhanced thermal responsiveness due to nanozyme incorporation (Figure S5, Supporting Information). In‐situ variable‐temperature atomic force microscopy (AFM) showed that Ru‐GOx‐PNN maintains a compact, ZIF‐like structure (≈70 nm height) at room temperature and expands to ≈85 nm at 42 °C, corresponding to volumetric swelling or structural rearrangement of the outer PNN thermosensitive hydrogel layer (Figure S6, Supporting Information). These results quantitatively illustrate the thermoresponsive “on/off” behavior of PNN.

The photothermal properties of the Ru‐GOx‐PNN nanozyme were then evaluated under 808 nm laser irradiation. The nanozyme exhibited concentration‐, power‐, and time‐dependent temperature elevation (Figure 1H,I; Figure S7, Supporting Information). Optimized conditions (100 µg mL^−1^ nanozyme concentration, 0.6 W cm^−2^ laser power, and 5 min irradiation) raised the solution temperature to approximately 42 °C, achieving the mild hyperthermia range required for mPTT while minimizing nonspecific thermal damage. Under these conditions, Ru–GOx–PNN displayed excellent thermal stability over five consecutive heating/cooling cycles, with negligible changes in peak temperature (Figure 1J). The photothermal conversion efficiency of the nanozyme was calculated as 41.29% based on the cooling profile (Figure 1K,L), highlighting its efficient light‐to‐heat conversion and its potential for photothermal interventions.

Post‐irradiation TEM imaging confirmed that Ru‐GOx‐PNN retained its dodecahedral morphology, even after hydrogel removal via centrifugation (Figure S8A, Supporting Information). DLS analysis revealed a decrease in hydrodynamic size to 247.00 ± 67.73 nm, comparable to Ru‐GOx, further supporting that photothermal heating effectively triggered PNN dissolution and exposed the active catalytic core (Figure S8B, C, Supporting Information). This temperature‐triggered activation was further supported by BCA assay before and after photothermal stimulation, which showed significantly increased GOx content after irradiation (Figure S9, Supporting Information).

Together, these results highlight the nanozyme's ability to combine stable photothermal heating with on‐demand catalytic activation, reinforcing its potential as a smart nanozyme system for precisely regulated catalysis and programmable therapeutic applications.

### 2.2 Ru‐GOx‐PNN Nanozymes Combined with mPTT Promoted Multienzyme Catalytic Activity

**Figure 2:**
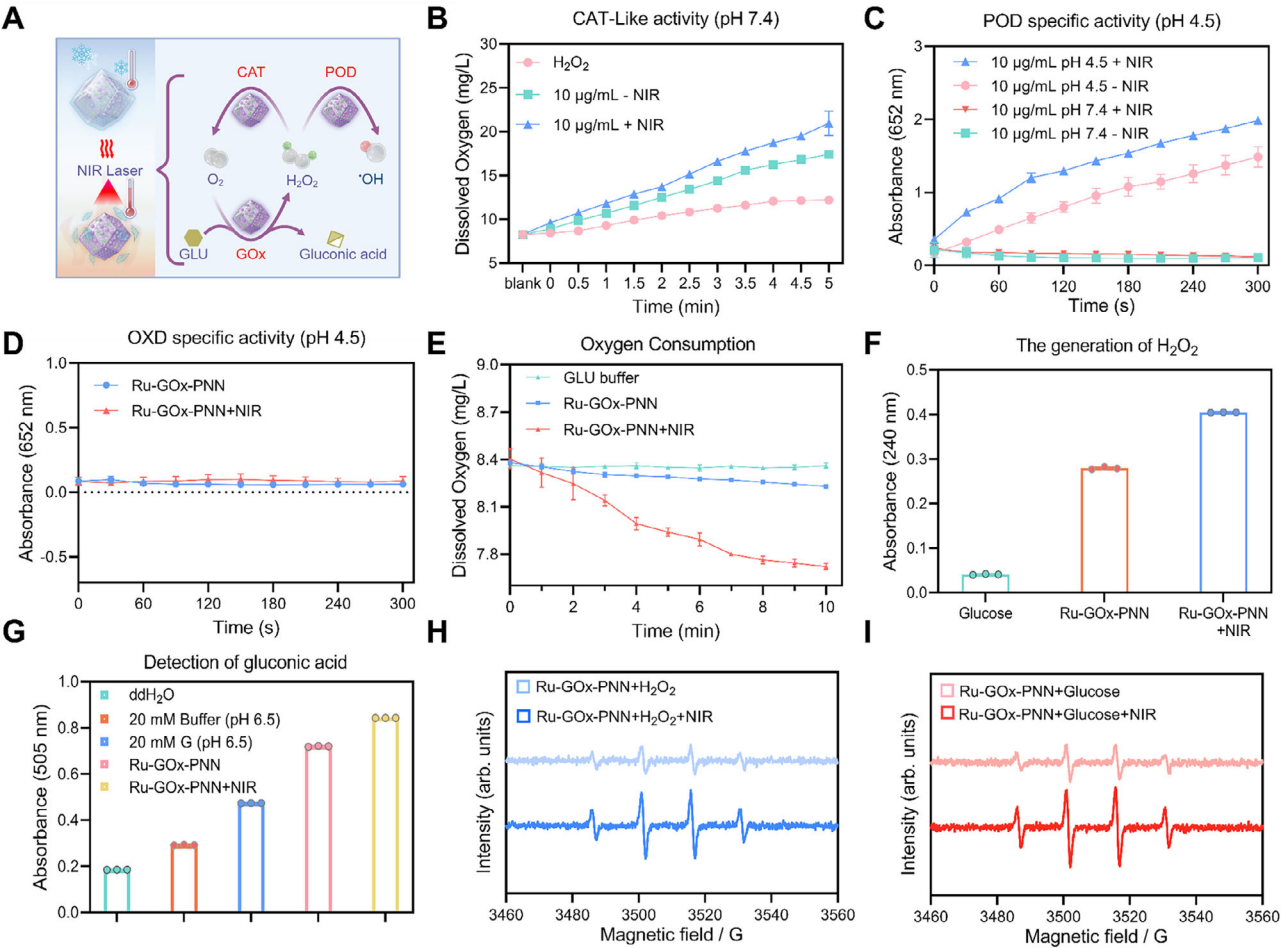
Evaluation of the multi‐enzyme catalytic activity of Ru‐GOx‐PNN nanozymes and verification of their photothermal‐enhanced enzyme function. A) Schematic illustration of the photothermal‐enhanced cascade catalytic reactions mediated by Ru‐GOx‐PNN nanozymes. Created in BioRender. Jiang, B. (2025). B) Detection of dissolved oxygen levels in 200 mM H_2_O_2_ solutions catalyzed by Ru‐GOx‐PNN nanozymes (10 µg·mL^−1^) with or without NIR irradiation (n = 3). C) POD‐like activity of Ru‐GOx‐PNN nanozymes (10 µg·mL^−1^) evaluated in a TMB‐H_2_O_2_ system under NIR or non‐NIR conditions (n = 3). D) OXD‐like activity of Ru‐GOx‐PNN nanozymes (10 µg·mL^−1^) assessed using the TMB system with or without NIR irradiation (n = 3). E) Oxygen consumption by Ru‐GOx‐PNN nanozymes in glucose solution with or without NIR irradiation (n = 3). F) H_2_O_2_ generation by Ru‐GOx‐PNN nanozymes in glucose solution under NIR or non‐NIR conditions, measured by absorbance at 240 nm (n = 3). G) Quantification of gluconic acid produced by Ru‐GOx‐PNN nanozymes in glucose solution through reaction with Fe^3+^ and hydroxylamine, monitored at 505 nm (n = 3). H) Detection of •OH radicals generated by Ru‐GOx‐PNN nanozymes in the presence of H_2_O_2_ with or without NIR irradiation using electron spin resonance (ESR) spectroscopy. I) Detection of •OH radicals produced by Ru‐GOx‐PNN nanozymes in the presence of glucose with or without NIR irradiation using ESR spectroscopy. Data were presented as mean ± SD.

To systematically evaluate the multienzyme activities of Ru‐GOx‐PNN nanozymes, we first investigated their intrinsic GOx, POD‐like, and CAT‐like activities, along with the regulatory effects of mild photothermal treatment (**Figure** **2**A). The enzymatic cascade activity was assessed under various reaction systems to determine the extent to which photothermal activation enhances catalytic performance.

As shown in Figure 2B, the Ru‐GOx‐PNN nanozymes catalyzed the decomposition of H_2_O_2_, leading to oxygen generation. This process was significantly augmented under NIR light exposure, indicating a photothermally enhanced CAT‐like activity. Similarly, in the TMB‐H_2_O_2_ reaction system, photothermal treatment significantly boosted the POD‐like catalytic efficiency (Figure 2C). Control experiments conducted in the absence of H_2_O_2_ showed negligible response, confirming that the signal originated from POD‐like catalysis rather than oxidase (OXD)‐like interference (Figure 2D).

The GOx activity was further verified by monitoring oxygen consumption, H_2_O_2_ generation, and gluconic acid production in glucose‐containing solutions. The results revealed substantial O_2_ consumption by Ru‐GOx‐PNN, which was further elevated under photothermal conditions, demonstrating thermally enhanced GOx activity (Figure 2E). Additionally, NIR irradiation significantly increased H_2_O_2_ production (Figure 2F). The subsequent generation of gluconic acid was also promoted, as evidenced by an increase in absorbance at 505 nm after complexation with Fe^3+^ and hydroxylamine (Figure 2G; Figure S10, Supporting Information), further supporting the increased GOx activity.

To further clarify the gating contribution of the PNN coating, we compared the catalytic performance of Ru‐GOx (without PNN) before and after photothermal heating (Figure S11, Supporting Information). Notably, Ru‐GOx exhibited only minor variations in CAT‐like, POD‐like, and GOx activities under the same photothermal conditions, indicating that the observed enhancement in Ru‐GOx‐PNN primarily arises from the thermoresponsive PNN layer. Combined with the temperature‐gradient results, these findings suggest that the PNN shell partially restricts substrate diffusion at ambient temperature but undergoes phase transition and swelling under mild photothermal stimulation, which markedly facilitates substrate accessibility and product release, thereby enabling intelligent and spatiotemporal control over catalytic activity. To evaluate its stability under physiologically relevant conditions, Ru‐GOx‐PNN was tested in phosphate‐buffered saline (PBS) and 10% fetal bovine serum (FBS)‐containing media. The nanozyme retained robust GOx, POD‐like, and CAT‐like activities (Figure S12A–E, Supporting Information) and maintained photothermal performance after repeated NIR irradiation (Figure S12F, Supporting Information), confirming excellent catalytic and photothermal stability under physiological conditions.

To further verify the generation of ROS during cascade catalysis, ·OH radical production was evaluated via ESR spectroscopy. In the presence of H_2_O_2_, Ru‐GOx‐PNN nanozymes generated prominent ·OH signals, which were further intensified under NIR irradiation, indicating enhanced POD‐like activity (Figure 2H). In glucose‐containing systems, significant ·OH generation was also detected, demonstrating the GOx‐POD cascade reaction, which was further promoted by photothermal treatment (Figure 2I). We then assessed dissolved oxygen generation in solutions containing GOx and H_2_O_2_ for Ru‐PMCS, GOx, and Ru‐GOx (Figure S13, Supporting Information). Ru‐PMCS exhibited enhanced oxygen production due to its intrinsic CAT‐like activity, while GOx alone consumed oxygen. Loading GOx onto Ru‐PMCS to form Ru‐GOx increased oxygen levels, though to a lesser extent than Ru‐PMCS alone, indicating that Ru‐GOx possesses dual CAT‐GOx cascade catalytic activities.

Taken together, these findings demonstrate that mild photothermal stimulation markedly enhances the multienzyme cascade activities of Ru‐GOx‐PNN nanozymes. This enhancement is mechanistically attributed to the thermoresponsive phase transition of the outer PNN shell, which dissolves upon heating to expose the catalytic Ru‐GOx core. The resulting exposure increases substrate accessibility and catalytic turnover, enabling precise spatiotemporal control of enzymatic activity. Furthermore, the coordinated GOx, POD‐like, and CAT‐like activities not only promoted efficient substrate conversion but also established an autocatalytic feedback loop, thereby extending the therapeutic potency. Collectively, these results underscore the potential of Ru‐GOx‐PNN nanozymes as a robust platform for spatiotemporally regulated catalytic therapy in precision medicine.

### 2.3 Ru‐GOx‐PNN Nanozymes Combined with mPTT Effectively Inhibit EC109 Cell Viability

**Figure 3:**
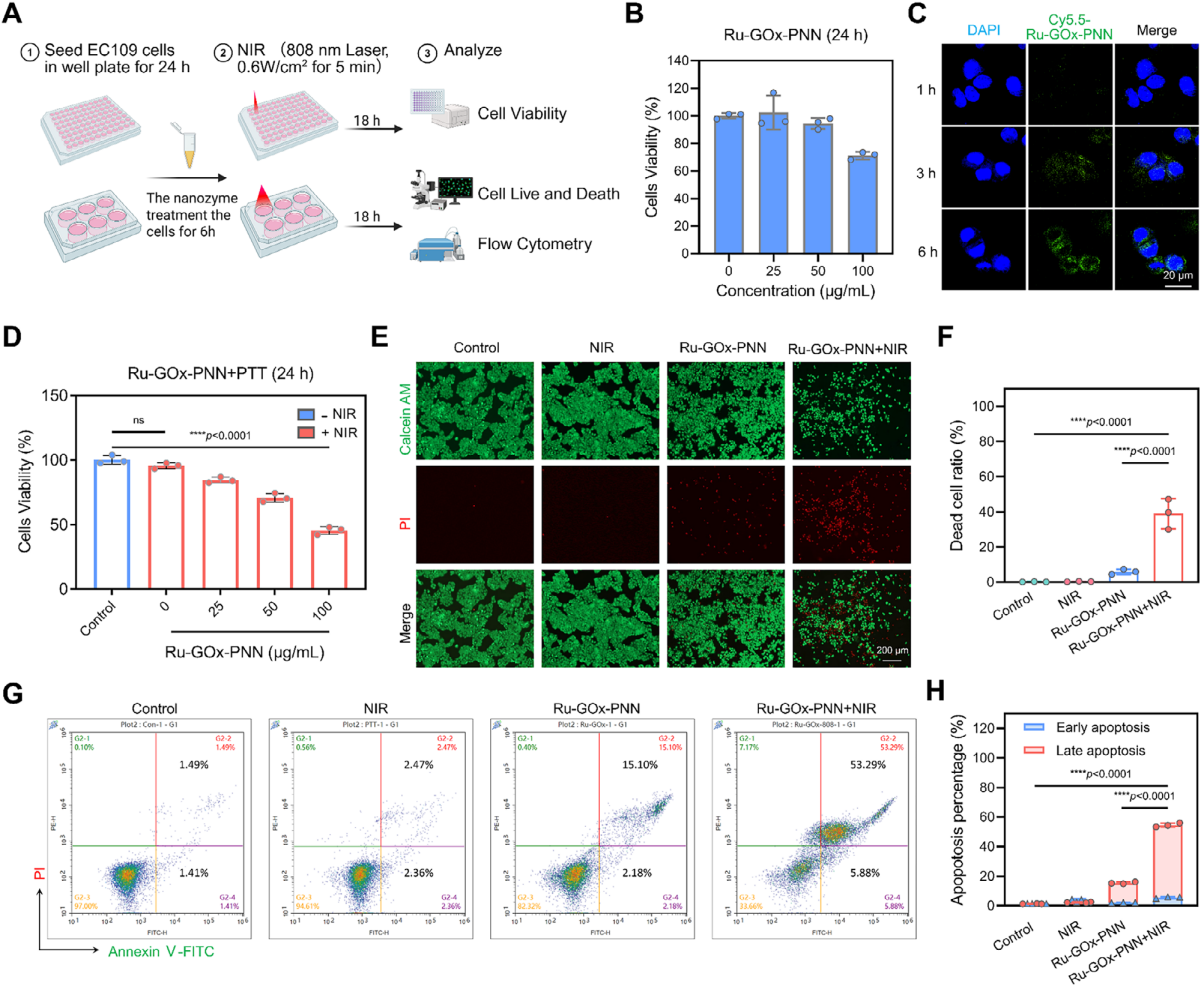
Ru‐GOx‐PNN nanozymes combined with mPTT inhibit EC109 cell viability. A) Schematic diagram of the in vitro study evaluating the inhibition of EC109 cell viability by Ru‐GOx‐PNN nanozymes combined with NIR irradiation. Created in BioRender. Jiang, B. (2025). B) Cell viability of EC109 cells treated with varying concentrations of Ru‐GOx‐PNN nanozymes (0‐100 µg·mL^−1^) for 24 h (n = 3). C) Cellular uptake of Cy5.5‐Ru‐GOx‐PNN nanozymes at different time points observed using CLSM. Scale bar = 20 µm. D) Cell viability of EC109 cells incubated with different concentrations of Ru‐GOx‐PNN nanozymes (0–100 µg·mL^−1^) combined with NIR irradiation (808 nm, 0.6 W·cm^−2^ for 5 min) for 24 h (n = 3, one‐way ANOVA with Tukey's multiple comparisons test). E, F) Calcein‐AM/PI co‐staining images and corresponding statistical analysis of EC109 cells treated with Ru‐GOx‐PNN nanozymes (100 µg·mL^−1^) with or without NIR irradiation (808 nm, 0.6 W·cm^−2^ for 5 min) (n = 3, one‐way ANOVA with Tukey's multiple comparisons test). Scale bar = 200 µm. G, H) Apoptosis of EC109 cells treated with Ru‐GOx‐PNN nanozymes (100 µg·mL^−1^) with or without NIR irradiation (808 nm, 0.6 W·cm^−2^ for 5 min), evaluated by flow cytometry and corresponding quantification (n = 3, one‐way ANOVA with Tukey's multiple comparisons test). Data were presented as mean ± SD.

The multi‐enzyme activities and photothermal properties of Ru‐GOx‐PNN nanozymes provide a robust basis for enhancing mPTT efficacy and suppressing tumor cell viability at the cellular level. To investigate this, a series of in vitro experiments were conducted to assess the inhibitory effects of Ru‐GOx‐PNN nanozymes combined with mPTT on human esophageal squamous carcinoma EC109 cells (**Figure** **3**A).

As shown in Figure 3B, treatment with varying concentrations of Ru‐GOx‐PNN nanozymes (0‐100 µg·mL^−1^) for 24 h did not significantly affect cell viability, suggesting minimal intrinsic cytotoxicity. Confocal laser scanning microscopy (CLSM) demonstrated time‐dependent cellular uptake of Cy5.5‐labeled nanozymes, with markedly enhanced at 6 h, which was selected as the incubation period for photothermal experiments (Figure 3C).

Upon NIR irradiation (808 nm, 0.6 W·cm^−2^ for 5 min), Ru‐GOx‐PNN nanozymes induced a pronounced and concentration‐dependent reduction in cell viability compared to laser treatment alone (Figure 3D). calcein acetoxymethyl ester (Calcein‐AM) / (propidium iodide) PI double staining corroborated these results, showing extensive cell death in the Ru‐GOx‐PNN + NIR group, whereas negligible cytotoxicity was detected in nanozyme‐treated groups without irradiation (Figure 3E,F). Additionally, flow cytometric analysis revealed a substantial increase in apoptotic cells following the combined treatment, underscoring the synergistic therapeutic outcome (Figure 3G,H).

Collectively, these in vitro results demonstrate that Ru‐GOx‐PNN nanozymes potently enhance mPTT‐mediated cytotoxicity against EC109 cells, underscoring their promise as an effective nanoplatform for synergistic cancer therapy.

### 2.4 Mechanistic Investigation of Ru‐GOx‐PNN Nanozyme‐Enhanced MPTT in vitro

Given that HSP70 is a central mediator of thermotolerance in tumor cells, we next examined how Ru‐GOx‐PNN nanozymes under mild photothermal conditions modulate HSP70 expression and associated stress pathways.

**Figure 4:**
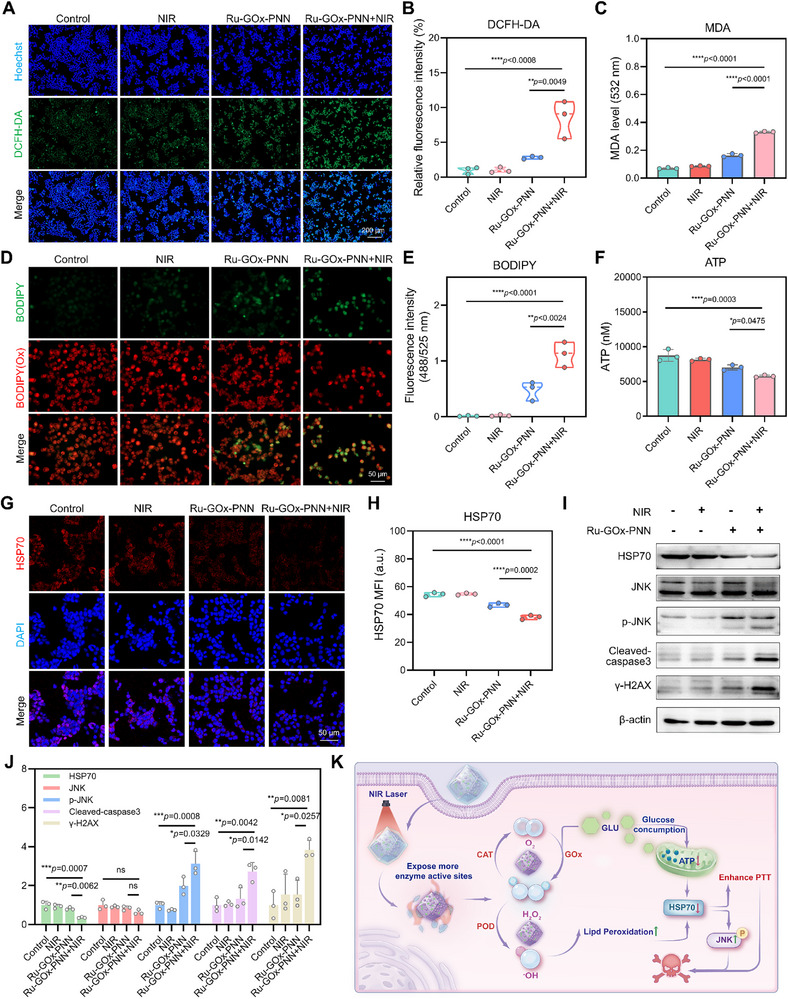
In vitro elucidation of mechanisms through which Ru‐GOx‐PNN nanozymes enhance mPTT. A) Intracellular ROS levels were assessed using the DCFH‐DA fluorescent probe (n = 3). Scale bar = 200 µm. B) Quantification of ROS fluorescence intensity (n = 3, one‐way ANOVA with Tukey's multiple comparisons test). C) LPO levels were measured using an MDA assay kit (n = 3, one‐way ANOVA with Tukey's multiple comparisons test). D) LPO was visualized using the C11‐BODIPY581/591+fluorescent probe. Scale bar = 50 µm. E) Quantification of LPO fluorescence intensity (n = 3, one‐way ANOVA with Tukey's multiple comparisons test). F) Intracellular ATP levels were determined following treatment with Ru‐GOx‐PNN nanozymes (100 µg·mL^−1^), with or without NIR irradiation (808 nm, 0.6 W·cm^−2^ for 5 min) (n = 3, one‐way ANOVA with Tukey's multiple comparisons test). G) HSP70 expression was evaluated by immunofluorescence staining in different treatment groups. Scale bar = 50 µm. H) Quantitative analysis of HSP70 fluorescence intensity (n = 3, one‐way ANOVA with Tukey's multiple comparisons test). I) Western blot analysis of HSP70, p‐JNK, cleaved caspase‐3, and γ‐H2AX expression in cells treated with Ru‐GOx‐PNN nanozymes (100 µg·mL^−1^), with or without NIR irradiation (808 nm, 0.6 W·cm^−2^ for 5 min). J) Densitometric quantification of Western blot bands (n = 3, one‐way ANOVA with Tukey's multiple comparisons test). K) The schematic diagram illustrates the synergistic mechanisms by which Ru‐GOx‐PNN nanozymes enhance mPTT. Data were presented as mean ± SD.

We first probed intracellular ROS generation using the fluorescent probe 2',7'‑ dichlorodihydrofluorescein diacetate (DCFH‐DA). As shown in **Figure** **4**A,B, Ru‐GOx‐PNN nanozymes combined with 808 nm irradiation markedly increased ROS levels. This enhancement is attributed to the intrinsic GOx‐POD cascade catalytic activity of the nanozyme system, glucose is oxidized by GOx to generate H_2_O_2_, which is subsequently decomposed into ·OH via POD‐like activity. The resulting ROS surge induced oxidative stress, thereby contributing to enhanced PTT efficacy. Given the critical role of ·OH in inducing lipid peroxidation (LPO), we assessed LPO levels by measuring malondialdehyde (MDA), a key LPO byproduct. The Ru‐GOx‐PNN + NIR group exhibited significantly elevated MDA content (Figure 4C). Consistently, fluorescence imaging with C11‐BODIPY581/591 confirmed intensified LPO in the combination treatment group (Figure 4D,E). These findings highlight LPO as a central mechanism of oxidative injury, with LPO‐derived aldehydes (e.g., MDA, 4‐HNE) known to destabilize HSP70 and promote its degradation under oxidative stress.^[^ ^10^, ^11^, ^41^ ^]^ Then, we performed co‐immunoprecipitation (Co‐IP) using an anti‐HSP70 antibody and probed for ubiquitin. The results showed that HSP70 ubiquitination was markedly increased in the Ru‐GOx‐PNN + PTT group compared with the control group (Figure S14A, Supporting Information). We also examined HSP70 expression following co‐treatment with the proteasome inhibitor MG132 or the autophagy inhibitor chloroquine. Western blot analysis revealed that MG132 partially restored HSP70 expression, whereas chloroquine had little effect (Figure S14B, Supporting Information). Together, these findings demonstrate that Ru‐GOx‐PNN + PTT triggers oxidative stress–mediated HSP70 ubiquitination and subsequent proteasomal degradation.

To investigate whether HSP70 function is suppressed due to impaired energy metabolism, we first measured intracellular ATP levels. As shown in Figure 4F, Ru‐GOx‐PNN combined with NIR irradiation significantly decreased cellular ATP content. This effect is likely attributed to GOx‐catalyzed glucose consumption, which directly depletes energy substrates. To validate this mechanism, we further evaluated the impact of Ru‐GOx without PNN encapsulation on cellular energy metabolism using the Seahorse assay. The results indicated that Ru‐GOx moderately impaired oxidative phosphorylation while more substantially suppressing glycolytic activity, providing direct evidence for ATP depletion (Figure S15, Supporting Information). Such an energy crisis is expected to compromise the function of the ATP‐dependent molecular chaperone HSP70. Consistently, subsequent immunofluorescence analysis (Figure 4G,H) revealed a marked downregulation of HSP70 expression following nanozyme‐mediated mild photothermal therapy. Collectively, these findings demonstrate that GOx‐driven ATP depletion and lipid peroxidation synergistically contribute to HSP70 suppression.

We further explored downstream signaling pathways. Western blot analysis revealed that suppression of HSP70 was accompanied by elevated levels of p‐JNK, together with increased expression of cleaved caspase‐3 and γ‐H2AX (Figure 4I,J). To confirm the role of HSP70, cells were co‐treated with the HSP70 agonist TRC051384. This co‐treatment partially reversed HSP70 suppression and attenuated the upregulation of cleaved caspase‐3 and γ‐H2AX in the Ru‐GOx‐PNN + NIR group (Figure S16, Supporting Information), indicating that HSP70 inhibition directly contributes to apoptosis induction and DNA damage under mild hyperthermia. These results indicate that loss of HSP70 chaperone activity permits JNK activation, which in turn promotes apoptosis and induces DNA damage under mild hyperthermia.

Taken together, Ru‐GOx‐PNN nanozymes markedly enhance the efficacy of mPTT through multiple synergistic mechanisms (Figure 4K). Upon 808 nm laser irradiation, additional catalytic sites are activated on the nanozymes, further amplifying their enzymatic activity and promoting tumor cell death via the following pathways. First, the GOx–CAT cascade depletes intracellular glucose and reduces ATP production, thereby downregulating HSP70 expression and impairing cellular thermotolerance. Second, the GOx‐POD cascade elevates ROS levels and induces lipid peroxidation, leading to oxidative destabilization of HSP70. Third, dual suppression of HSP70 facilitates JNK pathway activation, triggering apoptosis and DNA damage. Collectively, these synergistic effects collectively enhance mPTT efficacy by integrating photothermal ablation with nanozyme‐mediated biological regulation.

### 2.5 In Vivo Tumor Targeting and Photothermal Performance of Ru‐GOx‐PNN Nanozyme

**Figure 5:**
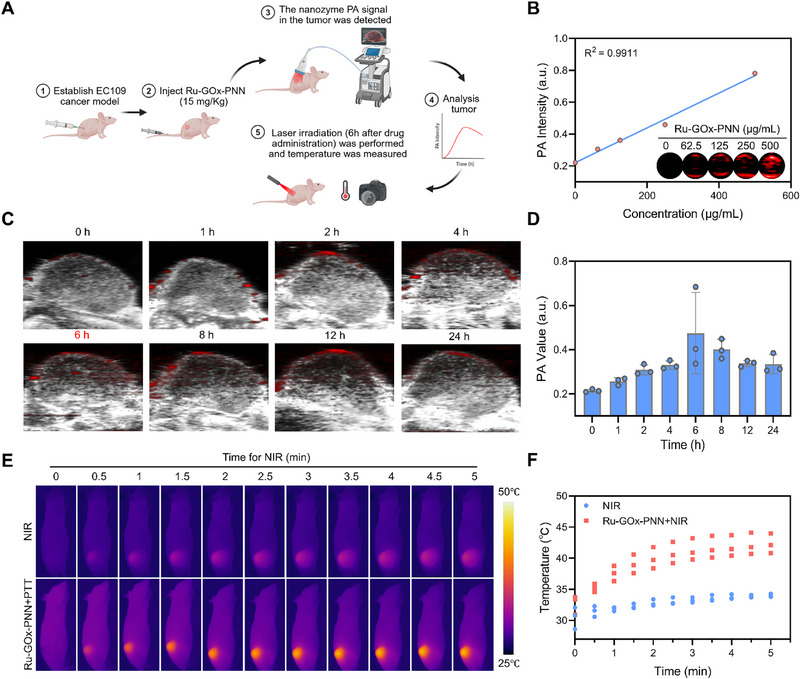
In vivo tumor targeting and photothermal performance of Ru‐GOx‐PNN nanozyme. A) Schematic illustration of the experimental protocol for in vivo PA imaging and photothermal effect validation of Ru‐GOx‐PNN. Created in BioRender. Jiang, B. (2025). B) in vitro PA signal intensities of Ru‐GOx‐PNN nanozymes at different concentrations, with corresponding PA images shown in the inset. C) Representative in vivo PA images of the tumor region in EC109 tumor‐bearing mice at various time points post‐intravenous injection of Ru‐GOx‐PNN. D) Quantitative analysis of average PA signal intensities in the tumor area at the indicated time points (n = 3). E) Infrared thermal images of EC109 tumor‐bearing mice following 808 nm laser irradiation (0.6 W·cm^−2^, 5 min) in different treatment groups. F) Quantitative analysis of temperature changes in the tumor region over time (n = 3). Data were presented as mean ± SD.

Encouraged by the in vitro findings, we next assessed the tumor‐targeting capability and photothermal effects of Ru‐GOx‐PNN nanozymes in vivo (**Figure** **5**A).

Initially, the photoacoustic (PA) imaging properties of Ru‐GOx‐PNN were assessed in vitro, which demonstrated a concentration‐dependent increase in PA signal intensity (Figure 5B), indicating the feasibility of using PA imaging as a non‐invasive tool to monitor in vivo nanozyme accumulation. Following intravenous administration of Ru‐GOx‐PNN (15 mg·kg^−1^) to tumor‐bearing mice, in vivo PA imaging revealed gradual accumulation of the nanozyme at the tumor site, with maximum signal intensity observed at 6 hours post‐injection (Figure 5C,D). This time point was therefore selected for subsequent photothermal irradiation.

To evaluate the photothermal conversion efficiency in vivo, mice were exposed to 808 nm laser irradiation (0.6 W·cm^−2^, 5 min) at 6 hours post‐injection. Infrared thermal imaging showed a rapid temperature increase in the tumors of the Ru‐GOx‐PNN + NIR group, reaching 42.30 ± 1.61 °C within 5 minutes (Figure 5E,F). In contrast, the tumor temperature in the NIR group exhibited only slight elevation, reaching 34.07 ± 0.25 °C, underscoring the essential role of nanozyme accumulation in achieving effective hyperthermia.

Together, these findings confirm the efficient tumor‐targeting capability and significant photothermal performance of Ru‐GOx‐PNN in vivo. The robust and localized temperature elevation achieved under mild irradiation conditions supports the potential of this nanozyme platform for spatially precise and safe photothermal ablation in therapeutic applications.

### 2.6 Ru‐GOx‐PNN Nanozyme‐Enhanced Antitumor Efficacy of MPTT

**Figure 6:**
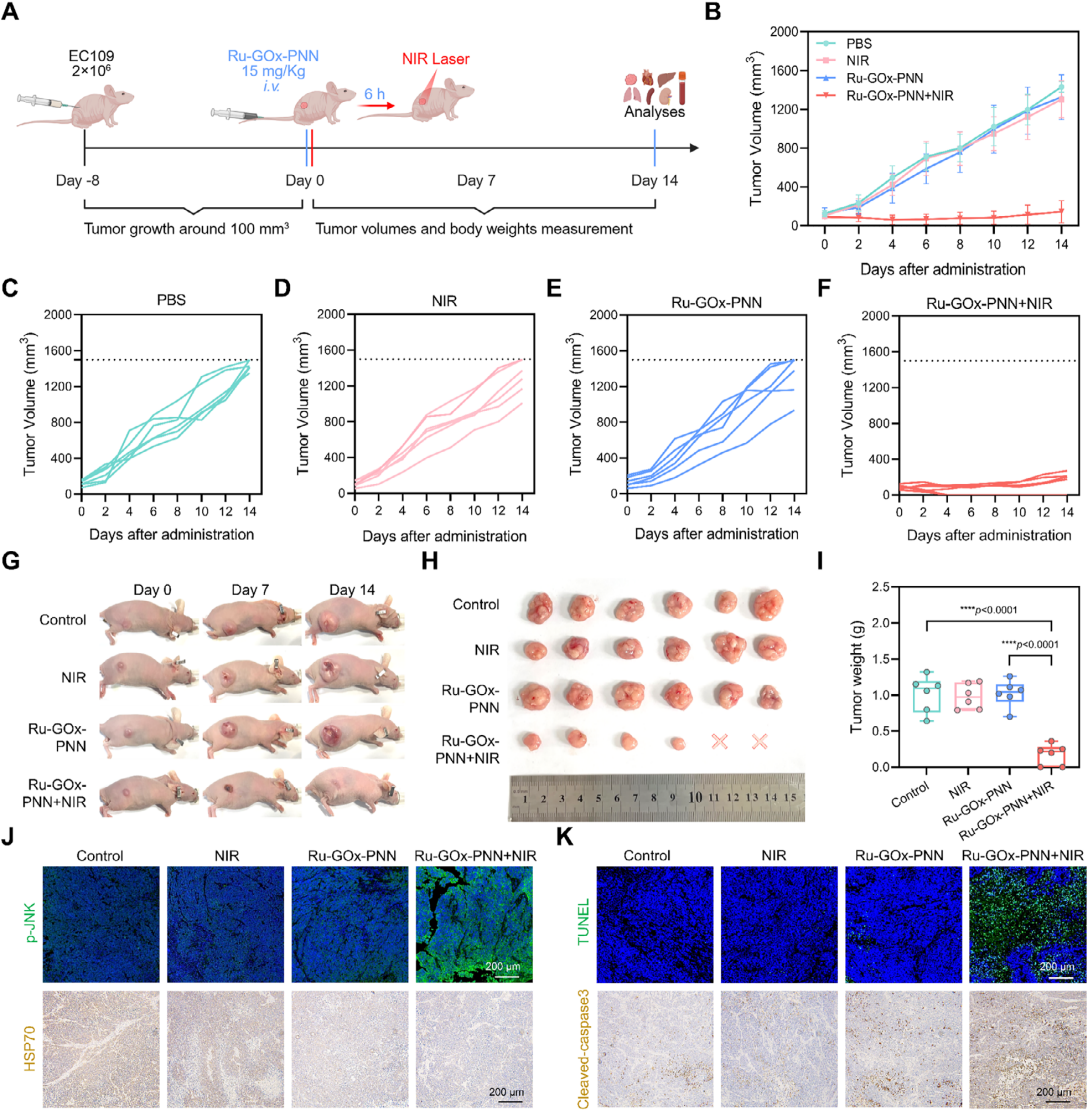
Anti‐tumor efficacy of Ru‐GOx‐PNN nanozyme combined with mPTT in EC109 tumor‐bearing mice. A) Schematic illustration of the treatment regimen for EC109 tumor‐bearing mice. Created in BioRender. Jiang, B. (2025). B) Tumor growth curves of mice in different treatment groups (n = 6). C–F) Individual tumor growth trajectories of mice in each treatment group (n = 6). G) Representative photographs of tumor‐bearing mice at days 0, 7, and 14 in each treatment group. H) Photographs of tumors excised from mice at the end of treatment. I) Quantitative analysis of tumor weights in different groups (n = 6, one‐way ANOVA with Tukey's multiple comparisons test). J, K) Representative immunofluorescence staining of p‐JNK and TUNEL, and immunohistochemical staining of HSP70 and cleaved caspase‐3 in tumor tissues from each group (n = 3, scale bar = 200 µm). Data were presented as mean ± SD.

To evaluate the in vivo antitumor efficacy of Ru‐GOx‐PNN nanozyme in combination with mPTT, a therapeutic study was conducted in EC109 tumor‐bearing mice. As illustrated in **Figure** **6**A, when tumor volumes reached approximately 100 mm^3^, mice were randomly assigned into four groups (n = 6 per group), (1) Control, (2) NIR (808 nm laser, 0.6 W·cm^−2^, 5 min), (3) Ru‐GOx‐PNN (15 mg·kg^−1^), and (4) Ru‐GOx‐PNN+NIR.

Throughout the 14‐day treatment period, both tumor volumes and body weights were monitored to assess therapeutic efficacy and systemic toxicity. The combination of Ru‐GOx‐PNN and NIR irradiation resulted in significant suppression of tumor growth compared to the control and all monotherapy groups (Figure 6B). Individual tumor growth curves (Figure 6C–F) and representative photographs of mice during the treatment course (Figure 6G; Figure S17, Supporting Information) further confirmed the enhanced therapeutic effect of the combined regimen. *Ex vivo* analysis of excised tumors at the endpoint showed visibly reduced tumor sizes and significantly lower tumor weights in the Ru‐GOx‐PNN + NIR group (Figure 6H,I), corroborating the pronounced antitumor efficacy achieved by the combination therapy.

To explore the molecular mechanisms underlying this enhanced effect, immunofluorescence and immunohistochemical staining were performed to assess the expression of HSP70 and p‐JNK. The Ru‐GOx‐PNN + NIR group exhibited substantial downregulation of HSP70 and concurrent upregulation of p‐JNK (Figure 6J; Figures S18, S19, Supporting Information), suggesting effective disruption of tumor cell self‐protection mechanisms. Furthermore, TUNEL staining and cleaved caspase‐3 immunohistochemistry revealed significantly increased apoptosis in tumors from the combination treatment group (Figure 6K; Figures S20, S21, Supporting Information), indicating robust activation of apoptotic pathways. Mechanistically, HSP70 downregulation may alleviate its suppression on JNK signaling, thereby promoting stress response activation and apoptosis induction. These in vivo observations align with previous in vitro findings, suggesting a shift in tumor cells from a protective to an apoptotic state under combination treatment.

In summary, the Ru‐GOx‐PNN nanozyme synergizes with mPTT to enhance antitumor efficacy through suppression of HSP70‐mediated thermoresistance and activation of JNK‐driven apoptosis. This dual mechanism not only improves therapeutic outcomes but also supports the potential of Ru‐GOx‐PNN as a safe and effective platform for precision cancer therapy.

### 2.7 Biosafety Evaluation of Ru‐GOx‐PNN Nanozyme Treatment

**Figure 7:**
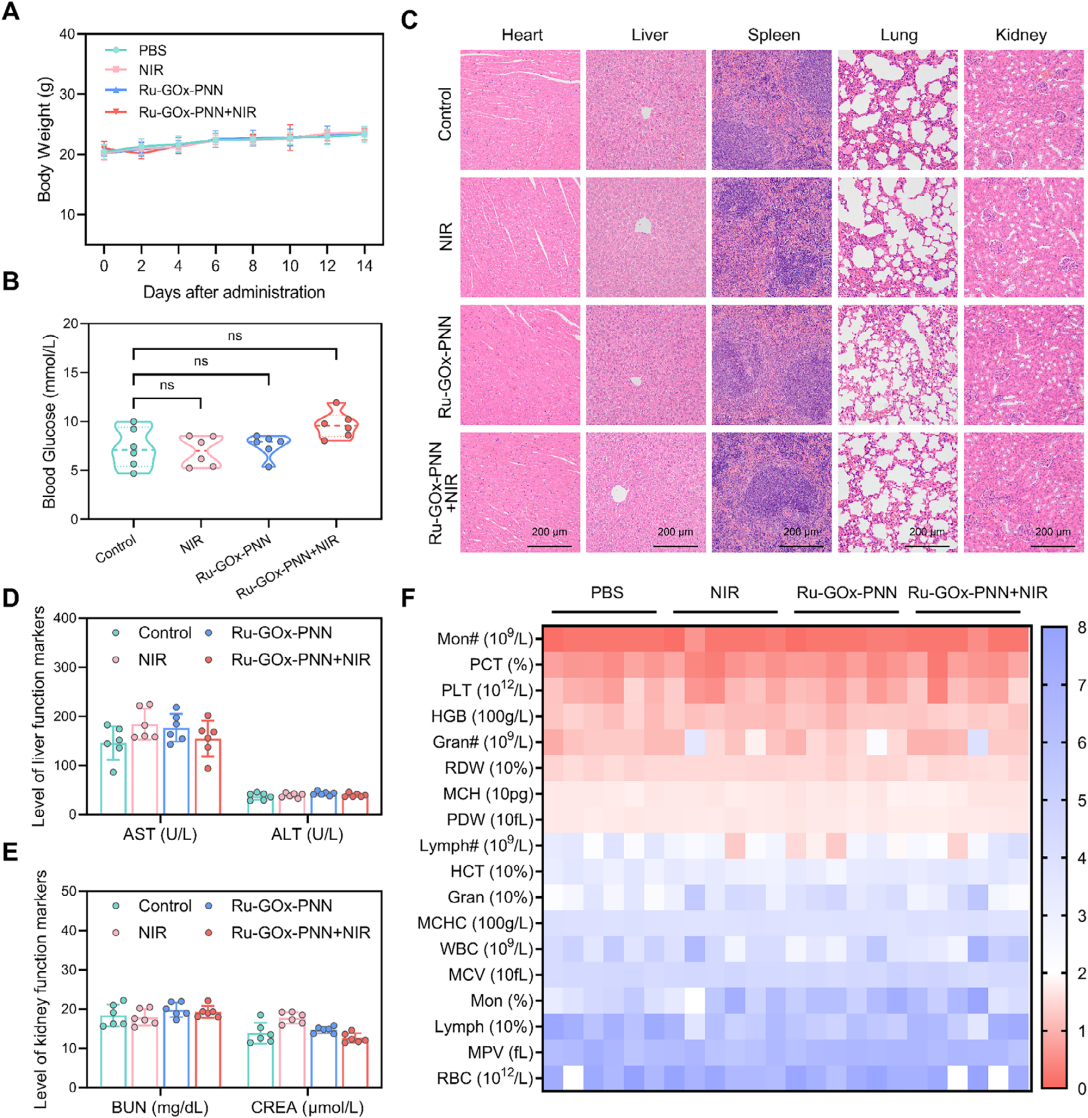
Biosafety analysis of Ru‐GOx‐PNN. A) Trend of body weight change during treatment in each experimental group (n = 6). B) On the 14th day of treatment, the changes of blood glucose of mice in different treatment groups (n = 6, one‐way ANOVA with Tukey's multiple comparisons test). C) Representative H&E staining images of the main organs (heart, liver, spleen, lung and kidney) of mice in each experimental group were collected after 14 days of treatment (n = 3). D, E) Serum biochemical indices, including liver function markers (AST and ALT) and kidney function markers (BUN and CREA), measured on the 14th day of treatment across all groups (n = 6). F) Blood routine parameters of all groups measured on the 14th day of treatment (n = 6). Data were presented as mean ± SD; ns, not significant.

To evaluate the biosafety of the Ru‐GOx‐PNN nanozyme in combined with mPTT in EC109 tumor‐bearing mice, body weight changes were monitored across all experimental groups during the 14‐day treatment period. Body weight measurements across all groups showed no significant loss, indicating the absence of obvious systemic toxicity (**Figure** **7**A).

Given the involvement of GOx in the treatment regimen, blood glucose levels were monitored to exclude potential metabolic disturbances. Results confirmed that glucose levels remained stable in all groups without statistically significant variations, supporting the metabolic safety and biocompatibility of the nanozyme‐based therapy (Figure 7B).

Histopathological analysis of major organs (heart, liver, spleen, lungs, and kidneys) via hematoxylin and eosin (H&E) staining revealed no evident structural abnormalities or tissue damage (Figure 7C). Furthermore, serum biochemical analysis indicated that liver function markers, aspartate aminotransferase (AST) and alanine aminotransferase (ALT), and renal function parameters, blood urea nitrogen (BUN) and creatinine (CREA), all fell within normal limits, suggesting no impairment of hepatic or kidney function (Figure 7D,E). Additionally, complete blood count parameters remained within physiological ranges across treatment groups, further affirming hematological safety (Figure 7F).

To evaluate the in vivo fate and clearance profile of Ru‐GOx‐PNN, we performed inductively coupled plasma mass spectrometry (ICP ‑ MS) analysis to quantify Ru levels in blood and fecal samples at different time points (1, 6, 12, and 24 h) post‐administration. As shown in Figure S22 (Supporting Information), the Ru content in blood decreased progressively over time, while fecal Ru levels exhibited an initial increase followed by a decline. Notably, Ru became nearly undetectable in both compartments by 24 h. These kinetic data demonstrate effective clearance of the nanozyme with minimal long‐term retention, further supporting the favorable biosafety profile of Ru‐GOx‐PNN after fulfilling its therapeutic function.

Together, these results demonstrate that the Ru‐GOx‐PNN nanozyme combined with mPTT exhibits a highly favorable biosafety profile in vivo, laying a solid foundation for its potential clinical translation in anticancer applications.

## 3 Conclusion

In this study, we developed a thermo‐responsive cascade catalytic nanozyme system (Ru‐GOx‐PNN) for precise and safe tumor therapy by overcoming HSP70‐mediated resistance. The nanozyme integrates ruthenium nanozyme and glucose oxidase into a phase‐transition polymeric network, enabling temperature‐controlled exposure of catalytic sites under NIR irradiation for switchable enzymatic activation.

Upon NIR irradiation, Ru‐GOx‐PNN initiates dual enzymatic cascades, the GOx‐POD cascade generates ·OH, inducing lipid peroxidation and facilitating HSP70 degradation, while the GOx‐CAT cascade depletes glucose and impairs ATP production, further suppressing HSP70 expression through disruption of energy metabolism. This dual inhibition of HSP70 not only attenuates tumor thermotolerance but also alleviates its suppression of the JNK signaling pathway, thereby augmenting apoptosis and potentiating antitumor efficacy.

In vitro studies confirmed the nanozyme's temperature‐dependent “on‐off” catalytic behavior and effective cascade amplification. In vivo experiments demonstrated that Ru‐GOx‐PNN combined with mild photothermal irradiation achieved efficient tumor suppression with minimal systemic toxicity, highlighting its potential for precise and biocompatible cancer treatment.

Collectively, this work presents a rationally designed nanozyme system that synergizes thermal responsiveness with cascade catalysis to achieve multimodal suppression of HSP70. The novelty of this strategy lies in its spatiotemporally controlled, self‐reinforcing mechanism, the thermo‐responsive gating ensures catalytic activity exclusively at the tumor site upon demand, thereby maximizing therapeutic specificity and minimizing off‐target effects. Meanwhile, the dual‐pathway cascade is ingeniously programmed not only to directly degrade HSP70 but also to disrupt its energy‐dependent expression, creating a feedback loop that comprehensively dismantles the key defense mechanism of tumors. This approach moves beyond conventional PTT or standalone nanozymes by integrating precise activation, energy metabolism interference, and thermoresistance ablation into a single, intelligent platform. Beyond the specific model explored, this work highlights the potential of thermally responsive cascade catalytic nanozymes to enable safe, precise, and efficient tumor therapy through intelligent microenvironmental modulation, providing useful insights for the future design and biomedical application of cascade catalytic nanozyme systems.

## 4 Experimental Section

### Materials and Reagents

Zn(NO_3_)_2_·6H_2_O (228 737) was obtained from Sigma‐Aldrich (St. Louis, MO, USA). Ru(acac)_3_ (0 102 3104) was purchased from Adamas‐beta (Shanghai, China). 2‐Methylimidazole (M813135), cetyltrimethylammonium bromide (H811117), tetraethyl orthosilicate (T819507), N‐isopropylacrylamide (N811777), and N,N,N″,N″‐tetramethylethylenediamine (N818999) were purchased from MACKLIN (Shanghai, China). NaOH (S580606), NaSH (S106641), N‐(Hydroxymethyl) acrylamide (N466614), N, N'‐Methylene‐bis‐acrylamide (M104027), and potassium persulfate (P292964) were received from Aladdin (Shanghai, China). GOx (G0050) was obtained from TCI (Shanghai, China). Antibodies used included HSP70 (Servicebio, GB11241; RRID, AB_3 714 562), JNK (Servicebio, GB114321; RRID, AB_3 714 563), p‐JNK (Cell Signaling technology, 9255; RRID, AB_2 307 321), cleaved‐caspase 3 (Cell Signaling technology, 9661; RRID, AB_2 341 188), and γ‐H2AX (Abcam, ab81299; RRID, AB_1 640 564). BCA protein assay kit (P0398), Calcein AM/PI cell cytotoxicity assay Kit (C2015L), 2 ′, 7 ′‐dichlorofluorescein diacetate (DCFH‐DA; S0033S), ATP assay kit (S0027), and MDA assay kit (S0131S) were purchased from Beyotime (Shanghai, China). The fluorescent probe C11BODIPY581/591 (BODIPY; D3861) was obtained from Thermo Fisher Scientific (Cleveland, OH, USA). Cell Counting Kit‐8 (CCK‐8; CA1210) and Annexin V‐FITC/PI Apoptosis Detection kit (CA1020) were purchased from Beijing Solarbio Science & Technology Co., Ltd.

### Synthesize of Ru‐ZIF‐8

Zn(NO_3_) _2_·6H_2_O (1.68 g) was dissolved in 80 mL methanol. Separately, 2‐methylimidazole (3.7 g) and Ru(acac)_3_ (0.7 g) were dissolved in 80 mL methanol. The two solutions were mixed and stirred vigorously for 24 hours. The products were collected by centrifugation, washed three times with methanol, and dried under vacuum at 100 °C for 24 hours. Next, the product was ultrasonically dispersed in 120 mL deionized water, followed by the addition of cetyltrimethylammonium bromide (6 mL, 25 mg mL^−1^), NaOH solution (10 mL, 6 mg mL^−1^), and tetraethyl orthosilicate (1.2 mL in 6 mL of methanol). The mixture was stirred for 2 h, washed with ethanol, and vacuum‐dried at 100 °C. The dried powder was calcined in a tube furnace, first heating to 300 °C for 2 hours, then to 900 °C for 5 hours, with a heating rate of 5 °C·min^−1^ under nitrogen atmosphere. Finally, the calcined product was dispersed in 6 M NaOH, stirred at 60 °C for 2 hours, and freeze‐dried to obtain Ru‐ZIF‐8.

### Synthesize of Ru‐GOx

Ru‐ZIF‐8 (5 mg) was dispersed into 2 mL of deionized water. GOx was added to the above solutions and stirred for 2 h, followed by the addition of NaSH (300 µL, 2 mM) solution and continued stirring for 2 h. The products were collected by centrifugation, washed by adding deionized water, and lyophilized under vacuum. NaSH plays a dual role during Ru–GOx nanozyme preparation, 1) as a coordination modulator, –SH groups bind with Ru and Zn–imidazolate sites to achieve uniform Ru dispersion and structural stabilization; and 2) as surface functional groups facilitating GOx immobilization through electrostatic and hydrogen‐bond interactions. GOx was non‐covalently anchored onto the Ru–ZIF‐8 surface via coordination and hydrogen bonding between enzyme residues and imidazolate ligands, preserving its bioactivity and ensuring stable integration.

### Synthesize of Ru‐GOx‐PNN Nanozyme

The PNN hydrogel was prepared according to the previous report.^[^ ^28^ ^]^ Briefly, 706.54 mg of N‐Isopropylacrylamide (NIPAm), 70.77 mg of N‐(Hydroxymethyl) acrylamide (NMAm), and 20 mg of N, N″‐Methylene‐bis‐acrylamide (BIS) were uniformly mixed and dispersed in deionized water (10 g) for 20 min, and then N_2_ was continuously introduced into the solution for 30 min to exclude oxygen. Potassium persulfate (K_2_S_2_O_8_) and N, N, N″, N′‐tetramethylethylenediamine (TEMEM) were then added to initiate polymerization, forming a poly(NIPAm)‐co‐NMAm hydrogel (PNN hydrogel). Subsequently, the PNN hydrogel was dialyzed using deionized water and freeze‐dried for 24 h. Then, the Ru‐GOx nanozymes were sonicated and dispersed in deionized water, and mixed with PNN at a 2:1 mass ratio. Finally, the mixture was centrifuged at 4 °C and lyophilized to obtain Ru‐GOx‐PNN nanozymes.

### Characterization

Morphological characterization of the nanozyme in aqueous solution was conducted using a Thermo Scientifi Talos L120C TEM at an operating voltage of 120 kV. DLS measurements were performed to analyze the size distribution of the materials dispersed in DDI water, using a Nano‐ZS90 dynamic light scattering instrument (Malvern, United Kingdom) at a concentration of 0.1 mg·mL^−1^. Elemental mapping analysis was used to determine the presence of Ru, C, N, and O in the nanozyme. XRD analysis was conducted to characterize the crystalline structures using a Bruker D8 Focus diffractometer (Germany) with Cu Kα radiation (λ = 1.5406 Å). Specific functional groups were identified using a Thermo Scientific Nicolet iS10 infrared spectrometer. The content and loading efficiency of GOx in the nanozyme were evaluated using a BCA protein assay kit. The decomposition of Ru‐GOx‐PNN nanozyme was investigated using thermogravimetric (TG) analysis.

### Detection of Photothermal Properties

Different concentrations of Ru‐GOx‐PNN nanozymes (0–400 µg·mL^−1^) were prepared and irradiated with 808 nm laser for 5 min with laser power of 0.6 W·cm^−2^. The heating process was photographed with an infrared thermal camera every 30 s to record the temperature. In addition, the temperature change of Ru‐GOx‐PNN nanozymes (100 µg·mL^−1^) was detected after 5 min irradiation with different laser power (0–1.5 W·cm^−2^).

To assess the photothermal stability, Ru‐GOx‐PNN solutions (100 µg·mL^−1^) were irradiated with an 808 nm laser (0.6 W·cm^−2^) for 5 minutes, followed by a cessation of irradiation to allow the solution to cool naturally to room temperature, representing one cycle. Five cycles were performed, and the photothermal cycling curve was recorded.

### Catalase‐Like Activity Assays

The catalase‐like activity was characterized by measuring the production of dissolved oxygen in the reaction system using a dissolved oxygen meter. The activity of the nanozymes was determined using a specific oxygen electrode on a multiparameter analyzer (JPBJ‐608, Leici, China) to monitor the increase in dissolved oxygen concentration. Ru‐GOx‐PNN nanozymes (10 µg·mL^−1^) were mixed with an aqueous H_2_O_2_ solution (200 mM), and the dissolved oxygen production in the solution was monitored. Additionally, the dissolved oxygen production was also measured after the nanozymes underwent photothermal treatment (0.6 W·cm^−2^ for 5 minutes).

### Peroxidase‐Like Activity and Oxidase‐Like Activity Assays

The peroxidase‐like activity of the nanozymes was evaluated using TMB as a substrate in the presence of H_2_O_2_. The nanozymes were first diluted to 100 µg·mL^−1^ with 50 mM phosphate buffer at pH 4.5 or 7.4. In a 96‐well plate, 10 µL of the nanozyme solution, 1 µL of TMB (20 mg·mL^−1^), and 5 µL of H_2_O_2_ (10 M) were sequentially added, followed by the addition of phosphate buffer to reach a final volume of 100 µL. The absorbance of TMB at 652 nm was measured using a microplate reader. In addition, the peroxidase activity was also compared before and after photothermal treatment (0.6 W·cm^−2^ for 5 min). Furthermore, the oxidase‐like activity of the nanozymes was assessed by conducting the experiment without adding H_2_O_2_ to the system.

### GOx‐Like Activity Assays

The GOx‐like activity of Ru‐GOx‐PNN nanozyme was evaluated by measuring O_2_ consumption, H_2_O_2_ production, and gluconic acid generation.^[^ ^29^ ^]^ Additionally, the effect of photothermal treatment (0.6 W·cm^−2^ for 5 min) on the O_2_ consumption, H_2_O_2_ generation, and gluconic acid production of Ru‐GOx‐PNN nanozyme was also assessed.

### Nanozymes‐Induced ·OH Production was Determined by ESR

ESR spectroscopy was performed at room temperature using a Bruker ESR spectrometer (A300‐10/12, Germany). DMPO was used as a ·OH scavenger. Using this method, the production of ·OH by Ru‐GOx‐PNN nanozymes dispersed in either H_2_O_2_ aqueous solution or glucose solution was compared. Additionally, the generation of ·OH by Ru‐GOx‐PNN nanozymes dispersed in different solutions before and after photothermal treatment was evaluated.

### Cell Culture

The human esophageal squamous cell carcinoma cell line EC109 (RRID, CVCL_6898) was purchased from Hunan Fenghui Biotechnology Co., Ltd. (Changsha, China) in December 2022. Authentication was conducted by short tandem repeat (STR) profiling at the Institute of Analysis and Testing, Beijing Academy of Science and Technology (Beijing Center for Physical and Chemical Analysis) on June 18, 2024, confirming a complete match with the reference profile of EC109 (RRID, CVCL_6898). Mycoplasma testing was performed by the same institute on June 18, 2024, and all results were negative. The cells were cultured in RPMI‐1640 medium, supplemented with 10% FBS and 1% penicillin/streptomycin to support cell growth and prevent bacterial contamination. Cells were routinely maintained at 37 °C in a humidified incubator with 5% CO_2_ to provide optimal conditions for proliferation. The medium was replaced every 2–3 days, and cells were subcultured when they reached 70–80% confluence.

### In Vitro Cytotoxicity

EC109 cells were seeded into 96‐well cell culture plates at a density of 5000 cells per well and incubated for 24 hours. The cells were then exposed to varying concentrations of Ru‐GOx‐PNN nanozymes (0–100 µg·mL^−1^) for an additional 24 hours. Cell viability was assessed using the CCK‐8 assay, following the manufacturer's instructions.

EC109 cells were seeded in 96‐well plates at a density of 5000 cells per well and incubated for 24 hours. The medium was then replaced with fresh medium containing various concentrations of Ru‐GOx‐PNN nanozymes (0–100 µg·mL^−1^). After 6 hours of incubation, the cells were exposed to 808 nm laser irradiation (0.6 W·cm^−2^) for 5 minutes and subsequently incubated for an additional 18 hours. Cell viability was then assessed using the CCK‐8 assay.

### Cellular Uptake Assay

EC109 cells were seeded in glass‐bottom cell culture dish (NEST Biotechnology, 801 001) and cultured for 24 hours. Cy5.5‐labeled Ru‐GOx‐PNN nanozymes were then added to the dishes and incubated for 1, 3, or 6 hours. Cellular uptake was visualized using confocal microscopy, with DAPI‐stained nuclei appearing blue and Cy5.5‐labeled Ru‐GOx‐PNN nanozymes appearing green.

### AM / PI Staining

EC109 cells were seeded in 6‐well plates and incubated for 24 hours. The cells were then divided into four treatment groups, (1) Control, (2) NIR (808 nm laser), (3) Ru‐GOx‐PNN, and (4) Ru‐GOx‐PNN+NIR. Ru‐GOx‐PNN nanozymes were used at a concentration of 100 µg·mL^−1^. After a 6‐hour incubation, cells in the NIR groups were subsequently exposed to 808 nm laser irradiation (0.6 W·cm^−2^) for 5 minutes, followed by an additional 18‐hour incubation. Cells were then stained with AM/PI for 30 minutes and observed under a fluorescence microscope.

### Apoptosis Assay

EC109 cells were seeded in 6‐well plates, and cells were divided into four groups as described in “AM/PI staining”. At the end of the nanozyme incubation, cells were harvested and treated with the Annexin V‐FITC/PI Apoptosis Detection kit before being analyzed for apoptosis by flow cytometry.

### Analysis of Intracellular ROS Level

The production of intracellular ROS was detected using a fluorescent probe DCFH‐DA (1:1000). EC109 cells were seeded in 6‐well plates and divided into four groups as described in the “AM/PI staining” method. After incubation with the nanozyme, cells from each group were collected, and diluted DCFH‐DA was added to each well for an additional 20 minutes of incubation to allow for staining. Following staining, excess dye was removed by washing with PBS. Fluorescence signals in EC109 cells were then observed using a fluorescence microscope to assess changes.

### Evaluation of Intracellular ATP Level

EC109 cells were seeded in 6‐well plates and divided into four groups as described in the “AM/PI staining” method. After nanozyme incubation, cells were collected, and ATP levels were measured using an ATP assay kit (Beyotime Biotechnology, S0027). Briefly, the medium was removed, and lysis buffer was added to each well, followed by cell disruption. After centrifugation at 12,000 rpm for 10 minutes, 20 µL of supernatant was mixed with 100 µL of ATP detection solution in a 96‐well plate, and luminescence was measured at 560 nm using a luminometer.

### Intracellular Lipid ROS Level Detection

EC109 cells were seeded in 6‐well plates and divided into four groups as described in the “AM/PI staining” method. EC109 cells were then stained with C11‐BODIPY581/591 in the dark at 37 °C for 30 min and finally visualized by fluorescence microscopy.

### Malondialdehyde Assay

EC109 cells were seeded in 6‐well plates and divided into four groups as described in the “AM/PI staining” method. EC109 cells were then treated according to the instructions provided by the MDA assay kit, and the levels of lipid peroxidation in the different treatment groups were measured.

### HSP70 Immunofluorescence Analysis

EC109 cells were seeded in in glass‐bottom cell culture dishs and divided into four treatment groups as described in the “AM/PI staining” method. After incubation, cells were washed with PBS and fixed with 4% paraformaldehyde for 10 minutes. Cells were then treated with 0.1% Triton X‐100 for 10 minutes and blocked with 5% BSA at room temperature for 1 hour. HSP70 antibody (1:200) was added for primary incubation, followed by staining with a fluorescent secondary antibody (Alexa Fluor 488). Finally, nuclei were counterstained with DAPI, and fluorescence images were captured using a laser scanning confocal microscope.

### Western Blot

EC109 cells were seeded in 10 cm culture dishes and grouped as described in the “AM/PI staining” method. After treatment, cells were collected, lysed, and protein concentrations were determined using a BCA assay. Equal amounts of denatured protein were separated by SDS‐PAGE and transferred onto PVDF membranes. The membranes were incubated with primary antibodies against HSP70 (1:1000), JNK (1:1000), p‐JNK (1:1000), cleaved‐caspase 3 (1:1000), and γ‐H2AX (1:1000), followed by secondary antibody incubation. Visualization was performed using ECL substrate and imaged on the MiniChemi 610 system. β‐Actin was used as a loading control.

### Establishment of Subcutaneous ESCC Tumor Model

All animal experiments were conducted in accordance with the ethical standards set by the Animal Ethics Committee of Zhengzhou University (approval number, ZZUIRBGZR2024‐1521). Six‐week‐old female BALB/c nude mice were obtained from SPF Biotechnology Co., Ltd. (Beijing, China) and housed according to the guidelines of the Animal Ethics Committee of Zhengzhou University. To establish a subcutaneous ESCC model, each mouse was implanted subcutaneously with 2 × 10^6^ EC109 cells in the right posterior flank. Tumor volume was calculated using caliper measurements of tumor length and width, according to the formula, V = Length × Width × Width /2.

### Photoacoustic Imaging Tracking

To evaluate the photoacoustic effect of Ru‐GOx‐PNN, solutions of varying concentrations (0‐500 µg·mL^−1^) were prepared and fixed in quartz tubes. PA signals were acquired using the Vevo LAZR‐X PA imaging system. For in vivo tumor accumulation studies, Ru‐GOx‐PNN (15 mg·kg^−1^) was intravenously injected into EC109 tumor‐bearing mice (n = 3). PA signals in the tumor region were recorded at various time points (1, 2, 4, 6, 8, and 24 h) post‐injection. The acquired data were analyzed using VevoLAB‐5.5.1 software to quantify changes in PA signals and assess the tumor accumulation of the nanozyme.

### In Vivo Photothermal Imaging

In vivo photothermal imaging of tumors was performed using an infrared thermal camera. When the tumor size reached approximately 100 mm^3^, PBS or Ru‐GOx‐PNN solution (15 mg·kg^−1^) was intravenously injected into EC109 tumor‐bearing mice (n = 3). Six hours post‐injection, tumors were irradiated with an 808 nm laser (0.6 W·cm^−2^) for 5 minutes. During the irradiation, thermal images were recorded at different time points to monitor the temperature change in the tumor region.

### In Vivo Antitumor Efficacy and Histological Assessment

EC109 tumor‐bearing mice were grouped into four treatment groups (n = 6), Control, NIR (808 nm, 0.6 W·cm^−2^ for 5 min), Ru‐GOx‐PNN (15 mg·kg^−1^), and Ru‐GOx‐PNN+NIR when tumor volume reached approximately 100 mm^3^. Ru‐GOx‐PNN nanozyme (15 mg·kg^−1^) was administered via intravenous injection. Six hours post‐injection, the tumor site was irradiated with an 808 nm laser at a power density of 0.6 W cm^−2^ for 5 minutes. Tumor volume was recorded every other day, with tumor volume calculated as, volume = (length × width2) / 2. Tumor volume changes were also recorded by photographing mice every 7 days during treatment. The study endpoint was reached when tumor volumes exceeded 1500 mm^3^, body weight loss exceeded 15%, or upon natural mortality.

On day 14 of treatment, all mice were euthanized, dissected, and tumors were collected. Tumor weight was recorded to assess treatment effect. The apoptosis of tumor cells was detected by terminal deoxynucleotidyl transferase dUTP Nick end labeling (TUNEL). The expressions of p‐JNK, HSP70 and cleaved‐caspase 3 in tumor tissues were evaluated by immunohistochemistry and Immunohistochemical fluorescence.

### Safety Assessment During Treatment

To assess the safety of the treatment, body weights of mice in each group were measured every other day during the treatment period. On day 14 of treatment, blood samples were collected for blood glucose measurement, complete blood count, and blood biochemical analysis.

### Statistical

Data were all expressed as mean ± standard deviation (SD). One‐way ANOVA with Tukey's multiple comparisons was used when comparing two or more group. Use GraphPad Prism 8.0 to compute all statistical differences. Values with *p*<0.05 were regarded as significant in all methods of statistical analysis.

## Conflict of Interest

The authors declare no conflict of interest.

## Supporting information



Supporting Information

Supporting Information

## Data Availability

The data that support the findings of this study are available in the supplementary material of this article.
