# Supplementary material for: Thermally Gated Dual‐Cascade Nanozyme for Enhanced Mild‐Temperature Photothermal Therapy
Source: Adv Sci (Weinh). 2025 Nov 7;13(18):e17528. doi: 10.1002/advs.202517528 (PMC13042585; doi:10.1002/advs.202517528)
Supplement: Supplementary file 1 — Supporting Information [file ADVS-13-e17528-s001.docx]

Supporting Information

**Thermally Gated Dual-Cascade Nanozyme for Enhanced Mild-Temperature Photothermal Therapy**

Shuyu Wang^1,2#^, Shenghui Wang^1#^, Mengyuan Cao^1^, lulu Wang^1^, Wei Jiang^2^, Xiyun Yan^1,3,4*^, Ying Liu^2*^, Bing Jiang^1,3*^

1. Nanozyme Laboratory in Zhongyuan, School of Basic Medical Sciences, Zhengzhou University, Zhengzhou, 450001, China.
2. National Health Commission Cardiovascular Disease Regenerative Medicine Research Key Laboratory, Central China Subcenter of National Center for Cardiovascular Diseases, Henan Cardiovascular Disease Center, Fuwai Central-China Cardiovascular Hospital, Central China Fuwai Hospital of Zhengzhou University, Zhengzhou 450046, China.
3. Nanozyme Laboratory in Zhongyuan, Henan Academy of Innovations in Medical Science, Zhengzhou, Henan, 451163, China.
4. CAS Engineering Laboratory for Nanozyme, Key Laboratory of Biomacromolecules, Institute of Biophysics, Chinese Academy of Sciences, Beijing 100101, China.

#These authors contributed equally to this work.

*Corresponding Authors:

Xiyun Yan, Email: yanxy@ibp.ac.cn;

Ying Liu, Email: fwhzliuying@zzu.edu.cn;

Bing Jiang, Email: [jiangbing@zzu.edu.cn](mailto:jiangbing@zzu.edu.cn)

**Supplementary Figures**


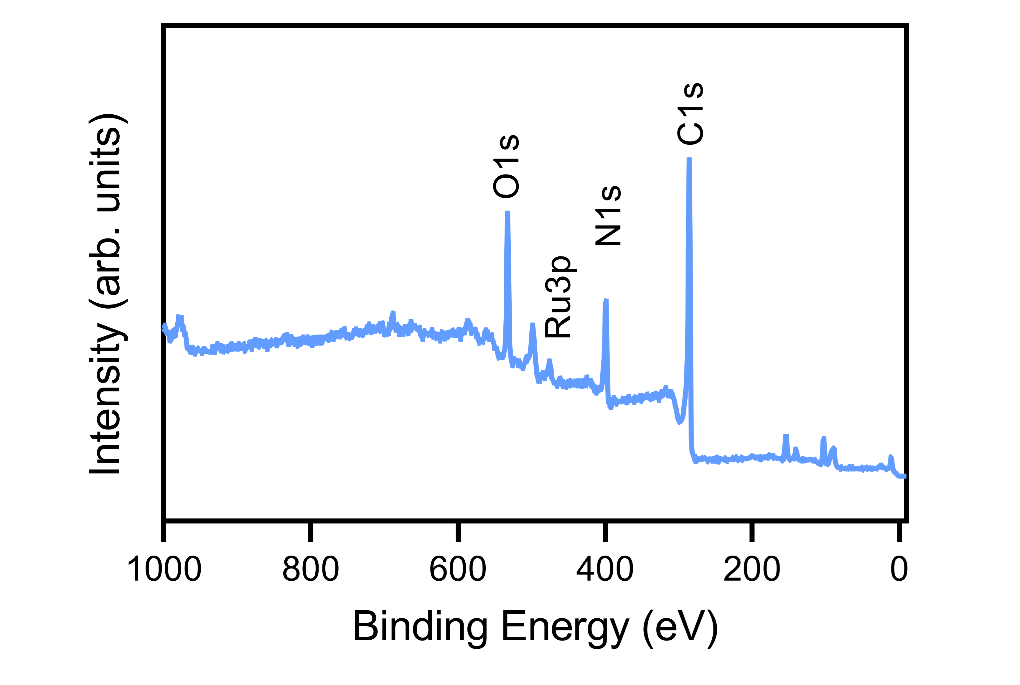


Figure S1. XPS characterization of Ru-GOx-PNN nanozymes.


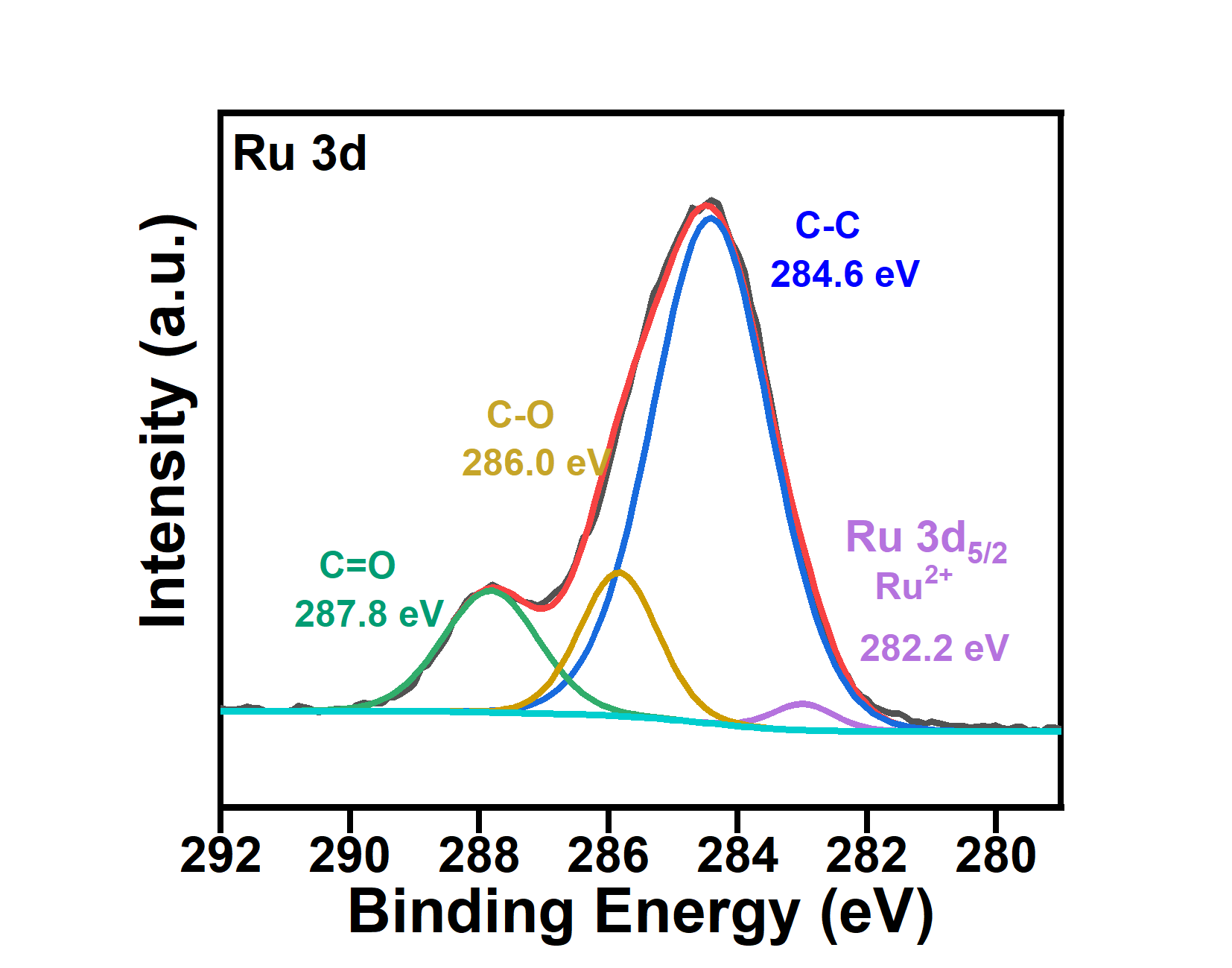


Figure S2. High-resolution XPS spectra of C1s + Ru3d of Ru-GOx-PNN nanozyme.


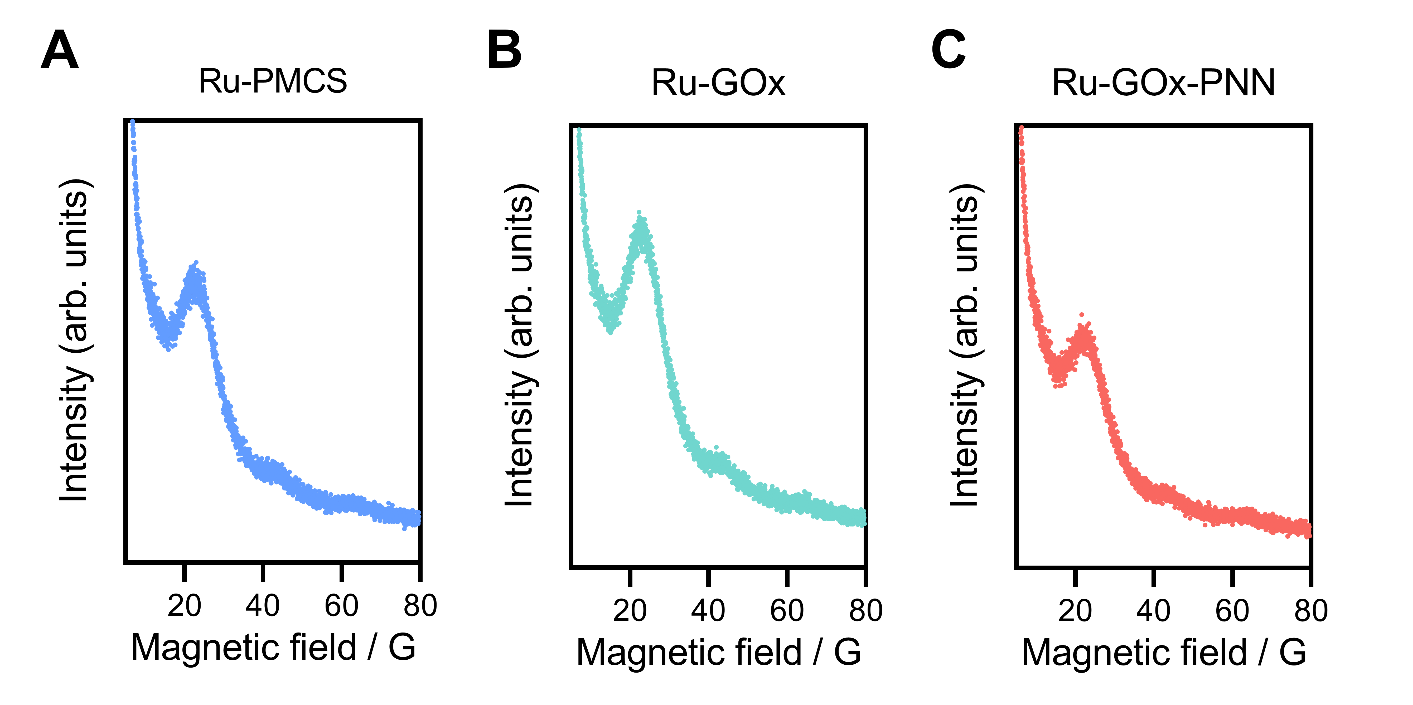


Figure S3. XRD patterns of Ru-PMCS (A), Ru-GOx (B), and Ru-GOX-PNN (C) nanozymes.


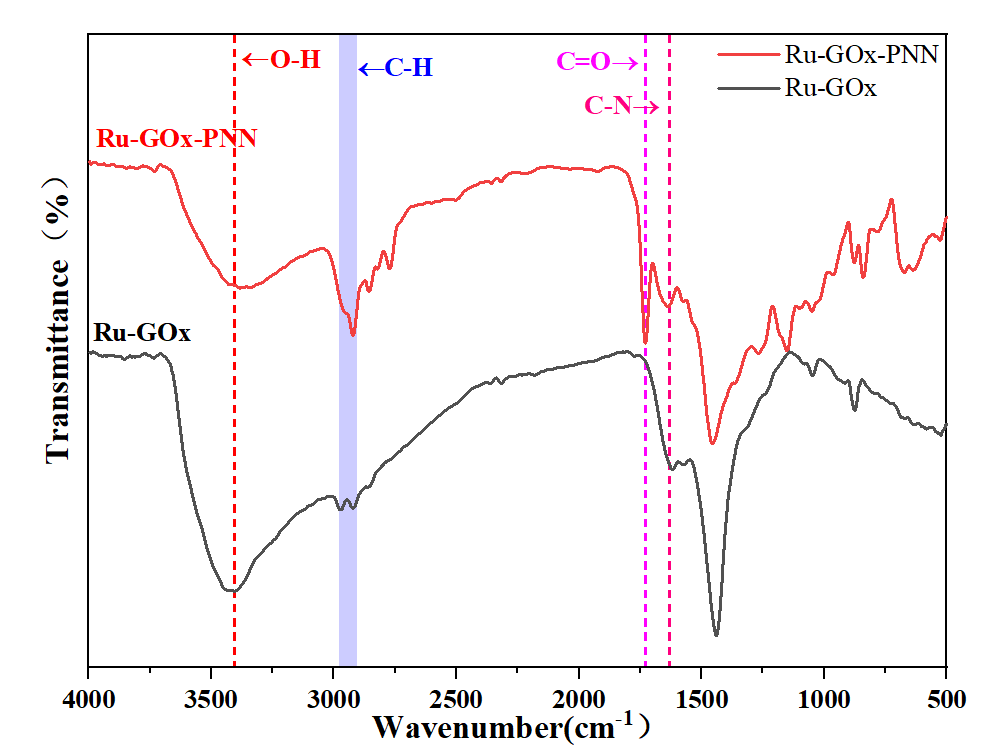


Figure S4. FTIR spectra of Ru-GOx and Ru-GOx-PNN nanozymes confirming the successful PNN encapsulation and preservation of GOx structural integrity.


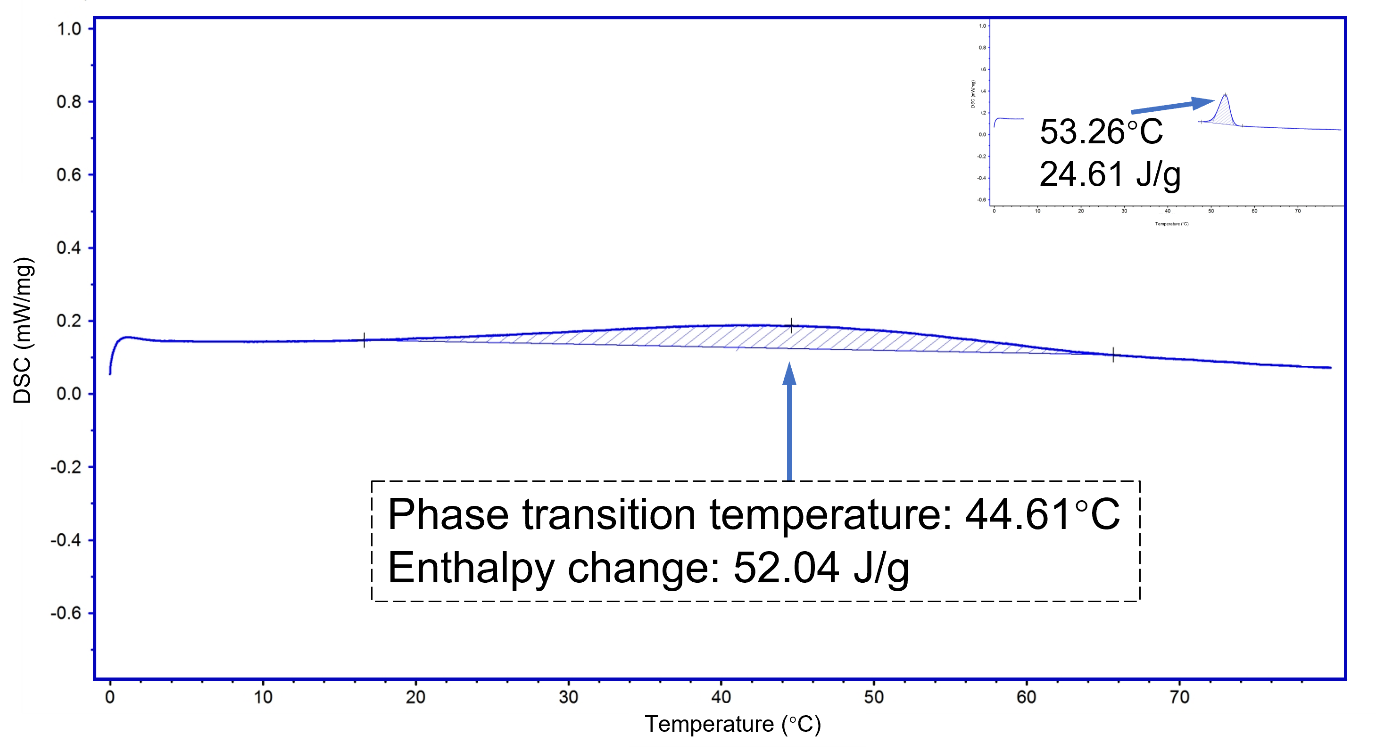
Figure S5. DSC curves of Ru-GOx-PNN nanozymes, with the inset showing the DSC curve of pure PNN.


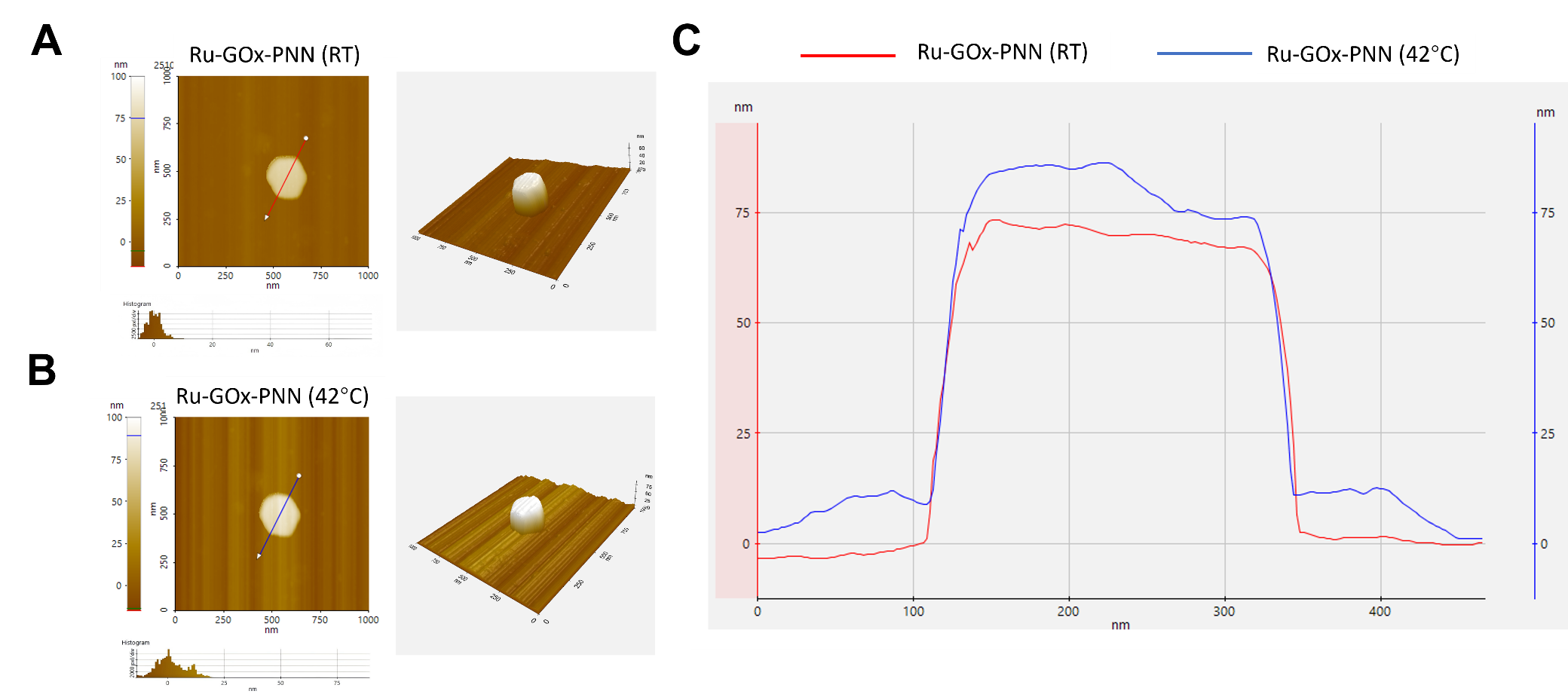
Figure S6. AFM images of Ru-GOx-PNN nanozymes. (A) 2D and 3D AFM images at room temperature. (B) 2D and 3D AFM images at 42 °C. (C) Cross-sectional height profiles at the same position of the nanozyme under different temperatures.


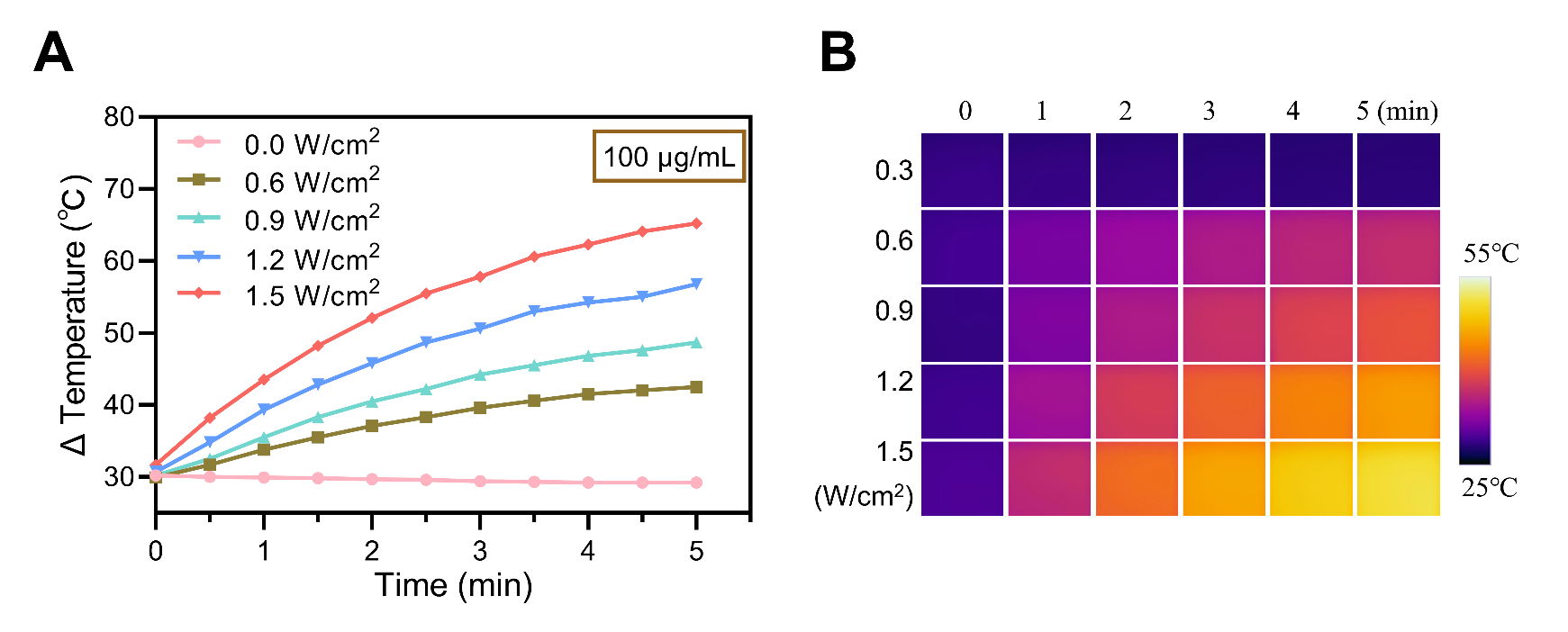


Figure S7. Photothermal Performance of Ru-GOx-PNN Nanozymes. A) Temperature elevation curves of Ru-GOx-PNN nanozymes (100 μg mL^-1^) under 808 nm laser irradiation at different power densities (0-1.5 W cm^-2^) for 5 min. B) Corresponding infrared thermal images of Ru-GOx-PNN nanozymes under the same conditions, illustrating power-dependent temperature increases.


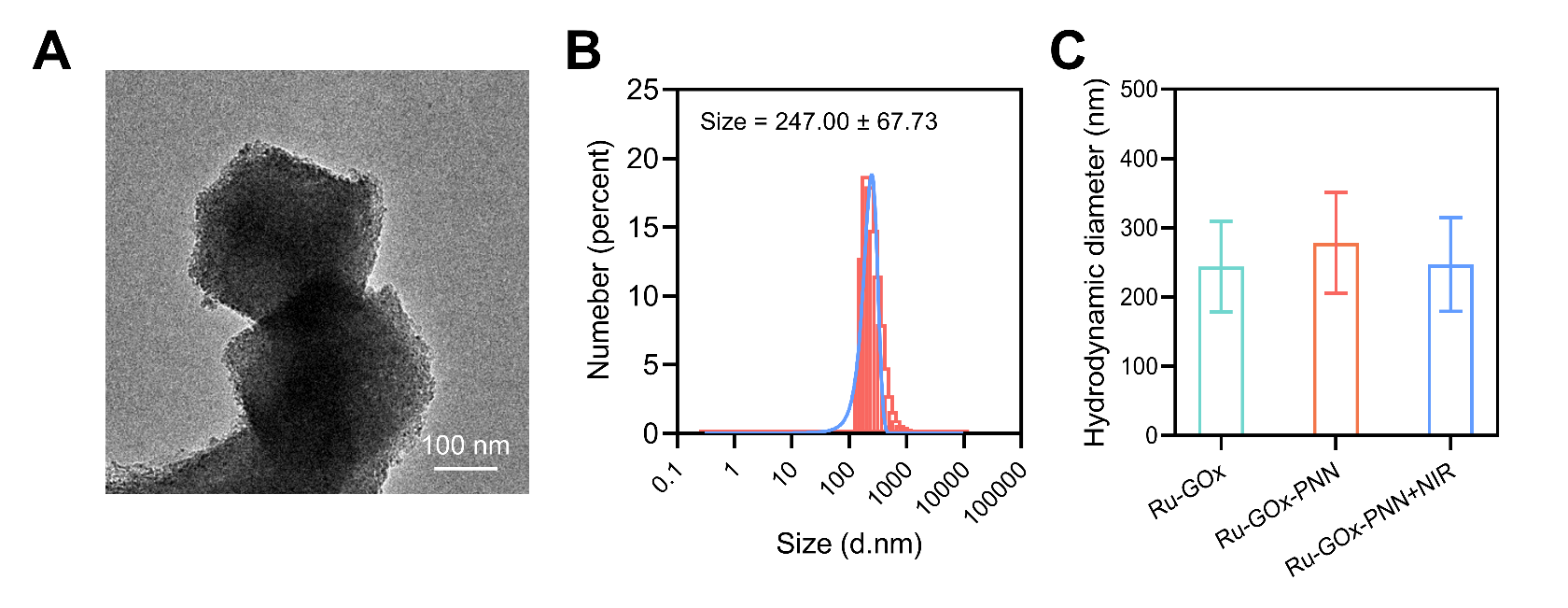


Figure S8. Post-Photothermal Characterization of Ru-GOx-PNN Nanozymes. A) TEM image of Ru-GOx-PNN nanozymes after 808 nm laser irradiation, showing preserved dodecahedral morphology. Scale bar = 100 nm. B) Hydrodynamic diameter of Ru-GOx-PNN nanozymes after photothermal treatment, as determined by DLS. C) Comparison of hydrodynamic diameters among Ru-GOx, Ru-GOx-PNN, and photothermally treated Ru-GOx-PNN nanozymes measured by DLS, confirming thermo-responsive size change.


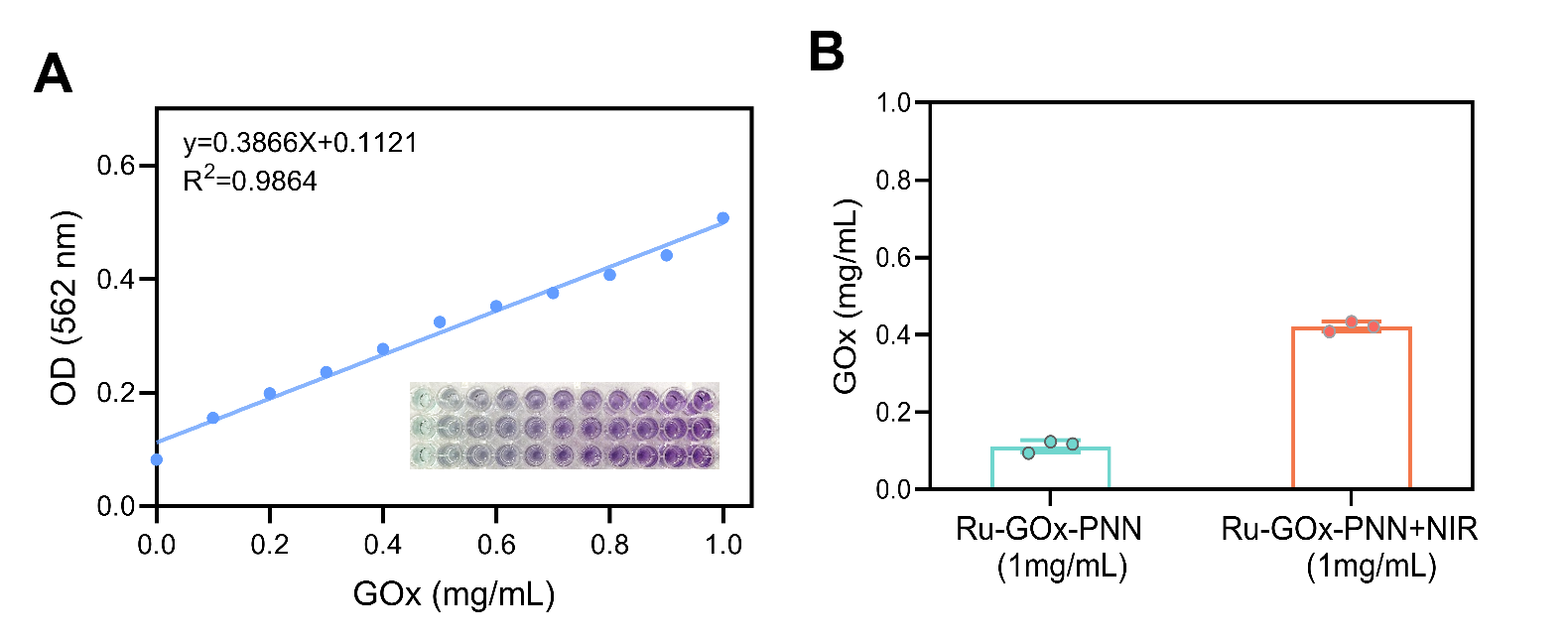


Figure S9. Quantitative Determination of GOx Content in Ru-GOx-PNN Nanozymes. A) Standard curve of GOx concentration established by the BCA protein assay. B) Quantitative analysis of GOx exposed from Ru-GOx-PNN nanozymes before and after photothermal treatment.


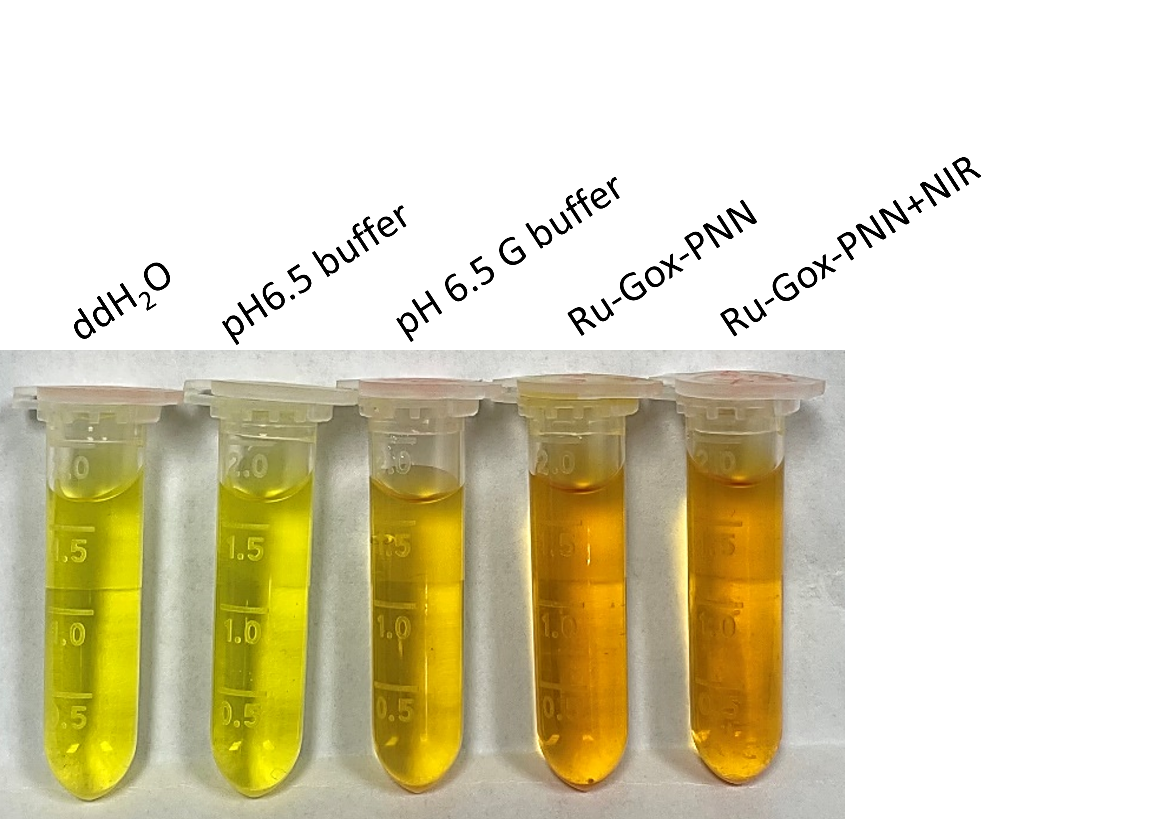


Figure S10. Representative images of red-brown compound formation from gluconic acid generated by Ru-GOx-PNN nanozymes in glucose solution reacting with Fe³⁺ and hydroxylamine.


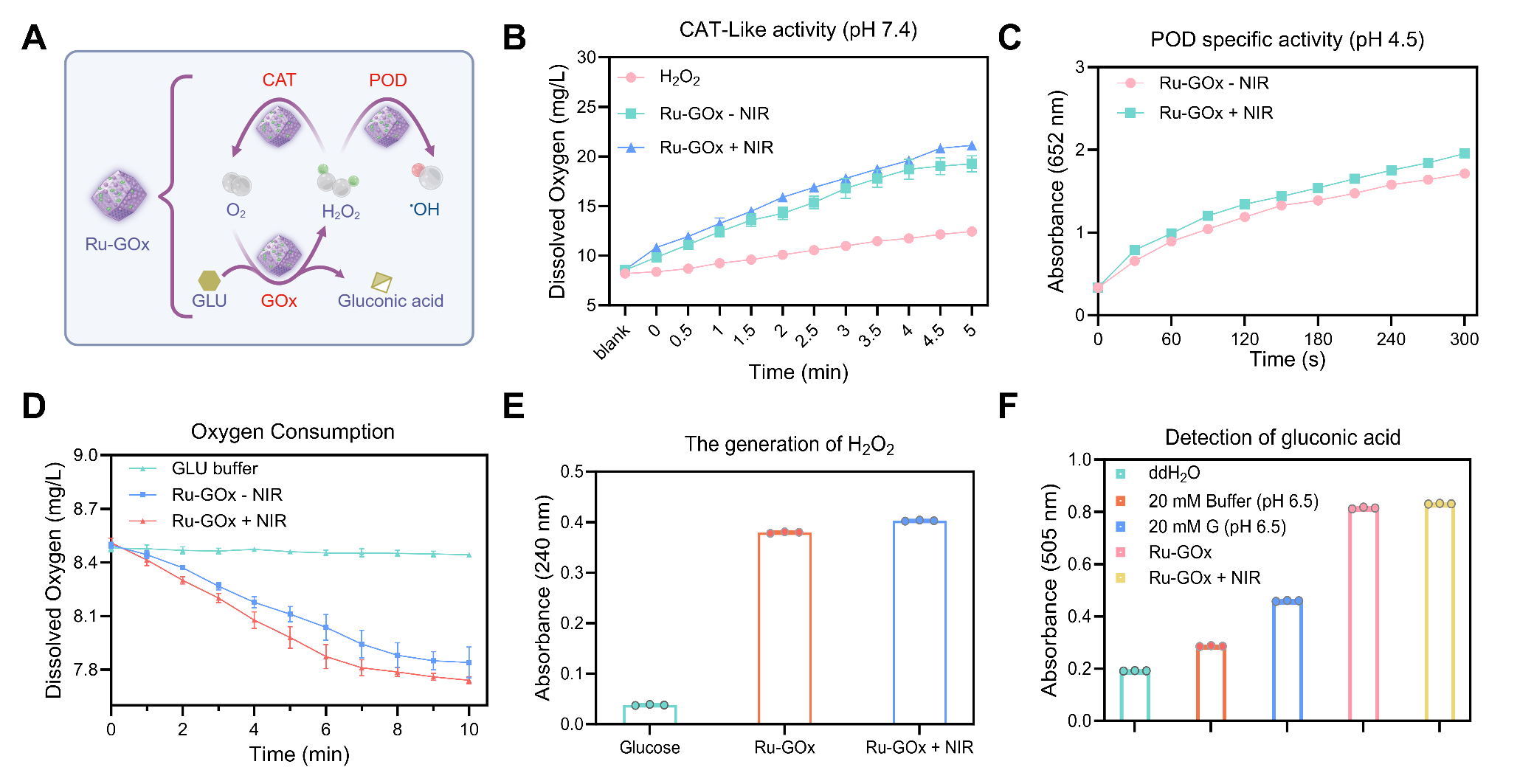


Figure S11. Evaluation of the multienzyme catalytic activities of Ru-GOx nanozymes before and after photothermal treatment. A) Schematic illustration of the cascade catalytic reactions mediated by Ru-GOx nanozymes. B) Detection of dissolved oxygen levels in 200 mM H_2_O_2_ solutions catalyzed by Ru-GOx nanozymes (10 μg·mL-1) with or without NIR irradiation (n = 3). C) POD-like activity of Ru-GOx nanozymes (10 μg·mL-1) evaluated in a TMB-H_2_O_2_ system under NIR or non-NIR conditions (n = 3). D) Oxygen consumption by Ru-GOx nanozymes in glucose solution with or without NIR irradiation (n = 3). E) H_2_O_2_ generation by Ru-GOx nanozymes in glucose solution under NIR or non-NIR conditions, measured by absorbance at 240 nm (n = 3). F) Quantification of gluconic acid produced by Ru-GOx nanozymes in glucose solution through reaction with Fe^3+^ and hydroxylamine, monitored at 505 nm (n = 3).


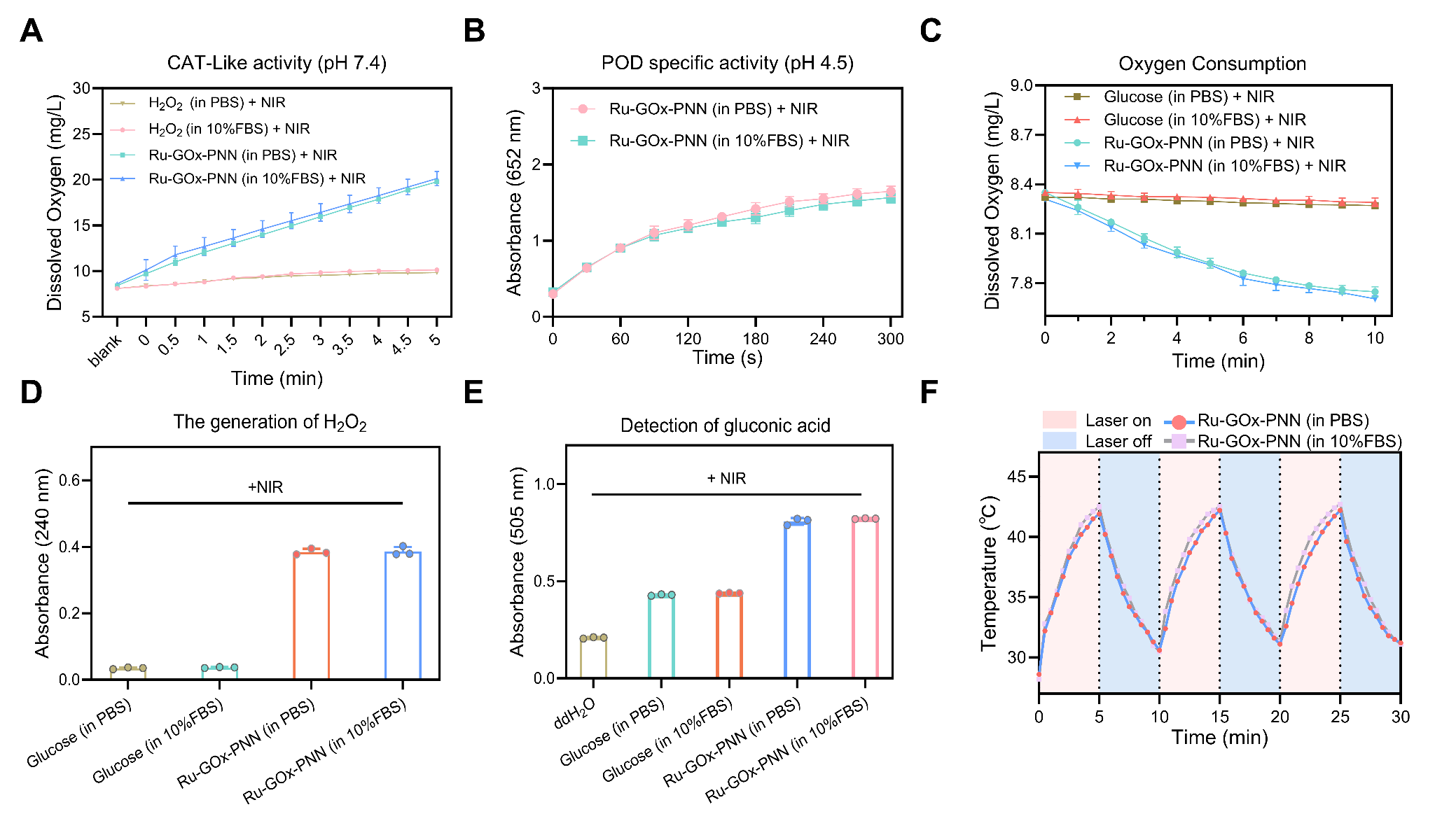
Figure S12. Evaluation of the multienzyme catalytic activities and photothermal stability of Ru-GOx-PNN nanozymes in PBS or 10% FBS-containing medium under NIR irradiation (808 nm, 0.6 W·cm^-2^). A) Dissolved oxygen levels in 200 mM H_2_O_2_ solution catalyzed by Ru-GOx-PNN nanozymes (10 μg·mL^-1^) with NIR irradiation (n = 3). B) POD-like catalytic activity of Ru-GOx-PNN nanozymes (10 μg·mL^-1^) in the TMB-H_2_O_2_ system under NIR irradiation (n = 3). C) Oxygen consumption in glucose solution catalyzed by Ru-GOx-PNN nanozymes with NIR irradiation (n = 3). D) Generation of H_2_O_2_ in glucose solution catalyzed by Ru-GOx-PNN nanozymes under NIR irradiation, monitored by absorbance at 240 nm (n = 3). E) Quantification of gluconic acid produced by Ru-GOx-PNN nanozymes in glucose solution via complexation with Fe^3+^ and hydroxylamine, monitored at 505 nm (n = 3). J) Photothermal stability of Ru-GOx-PNN nanozymes during multiple on/off laser irradiation cycles (808 nm, 0.6 W·cm^-2^).


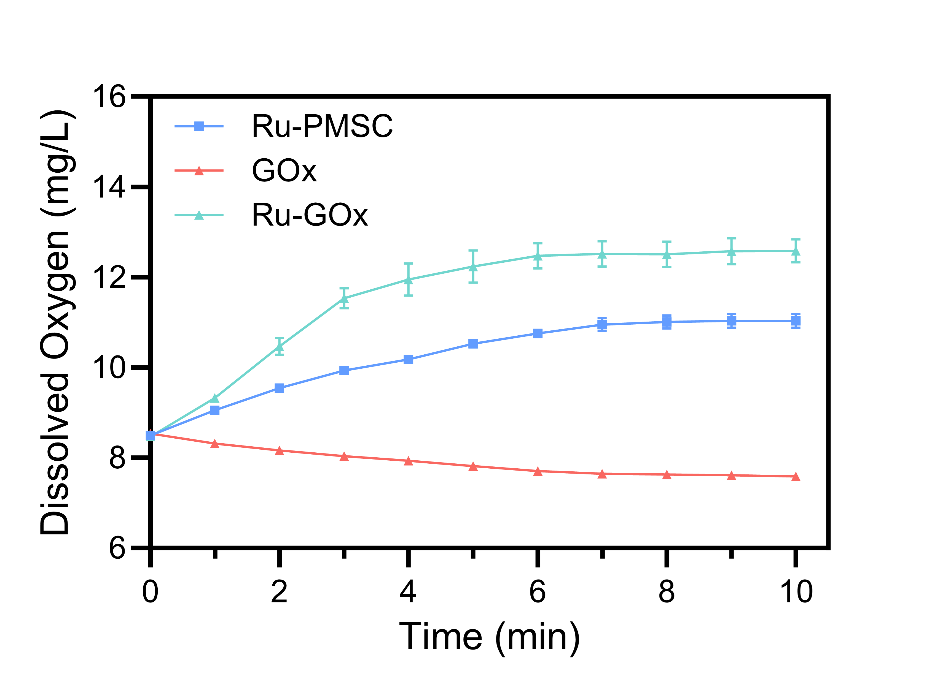
Figure S13. Dissolved oxygen generation or consumption by Ru-PMCS, GOx, and Ru-GOx in GOx (20 mM)-H_2_O_2_ (20 mM) solutions.


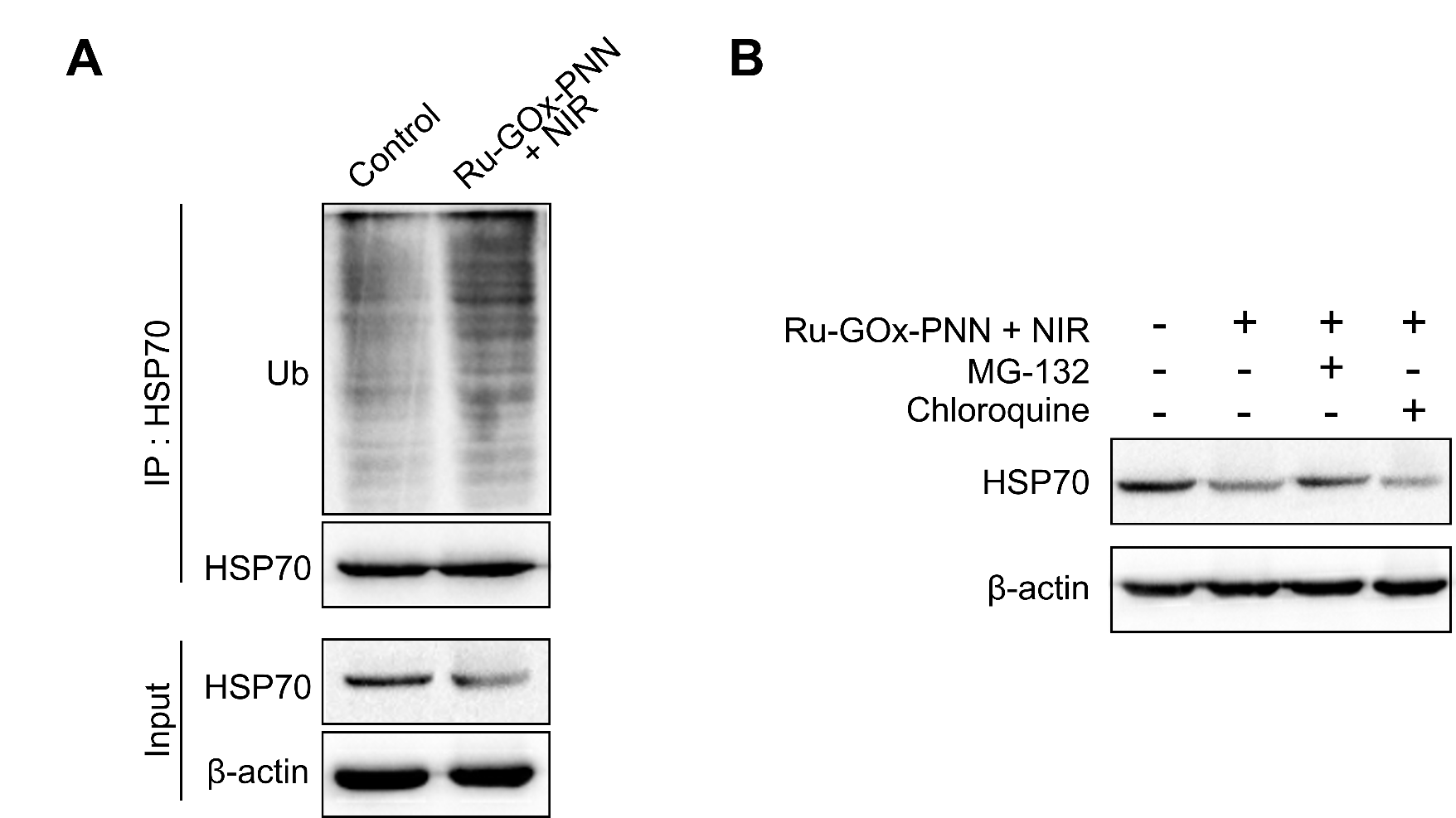
Figure S14. Analysis of HSP70 degradation pathway. A) Co-immunoprecipitation (Co-IP) of HSP70 and ubiquitin in different treatment groups. B) Western blot analysis of HSP70 expression after treatment with proteasome inhibitor MG132 or autophagy inhibitor chloroquine.


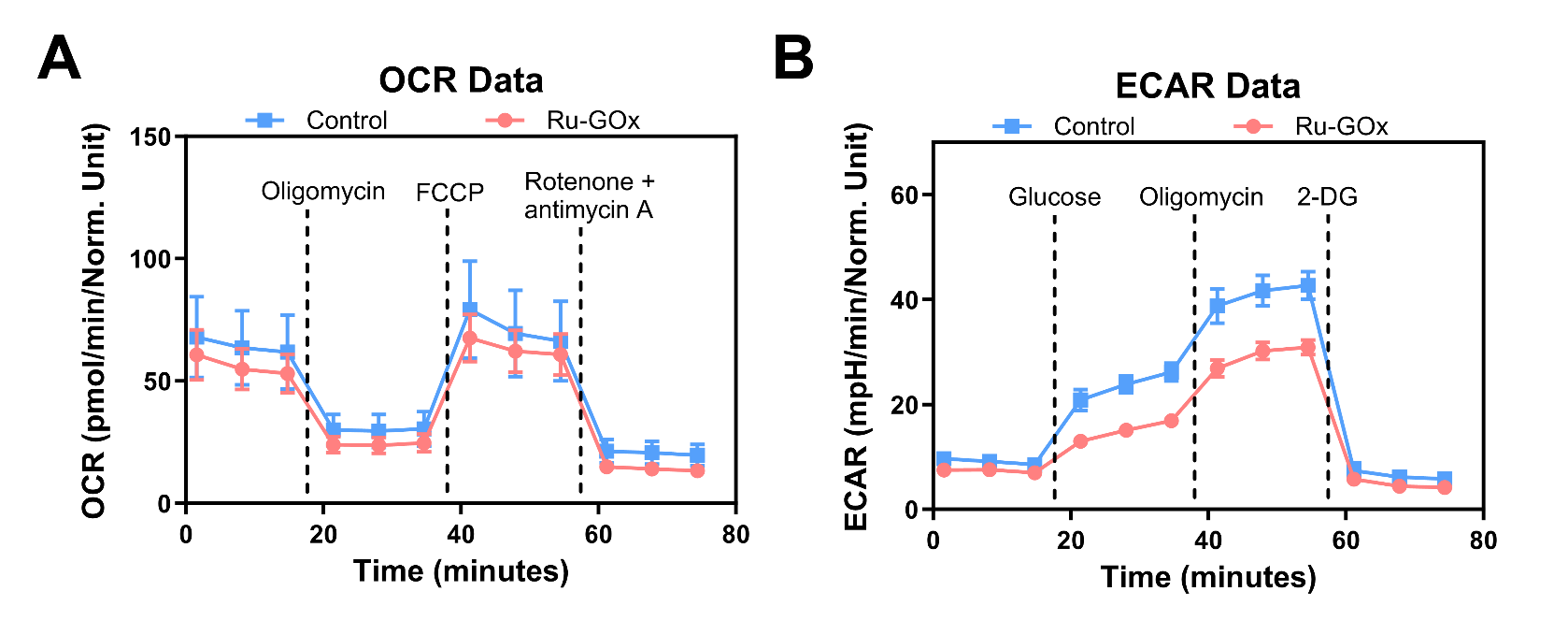
Figure S15. Seahorse analysis of Ru-GOx. A) Mitochondrial stress test showing oxygen consumption rate (OCR). B) Glycolysis stress test showing extracellular acidification rate (ECAR).


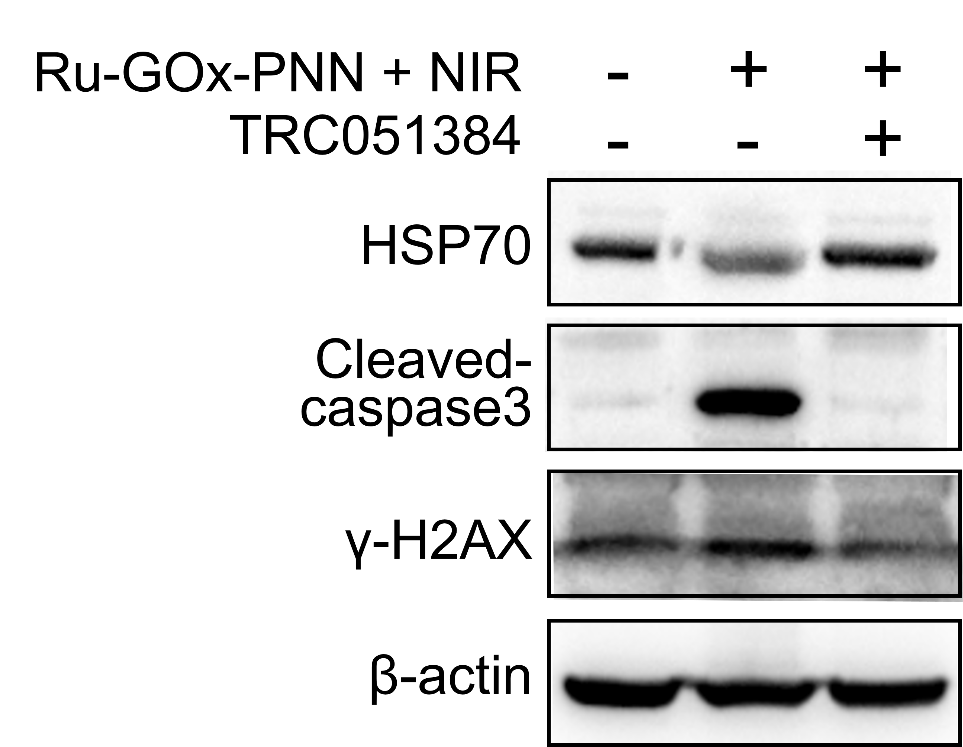
Figure S16. Effects of HSP70 activation on Ru-GOx-PNN + PTT induced apoptosis and DNA damage. Cells were co-treated with the HSP70 agonist TRC051384, and protein levels of HSP70, cleaved caspase-3, and γ-H2AX were assessed by Western blot.


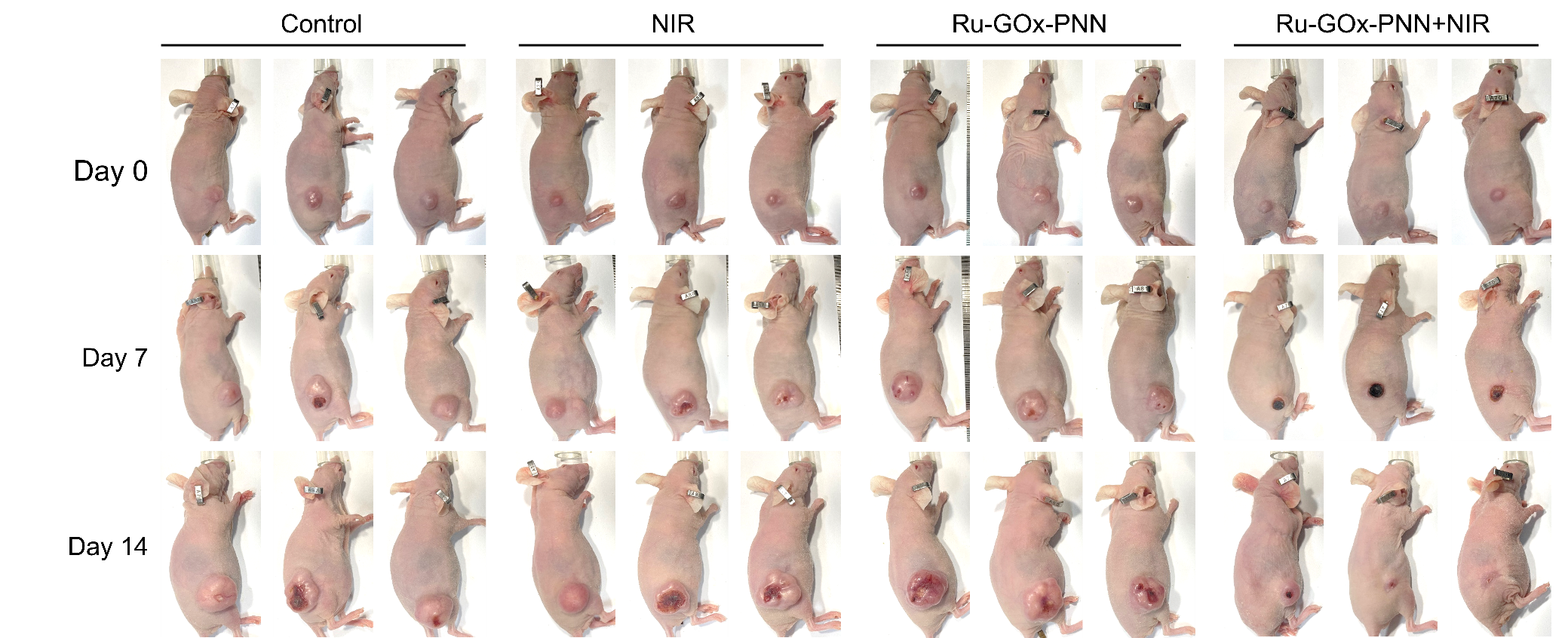


Figure S17. Representative images of EC109 tumor-bearing mice from each treatment group on days 0, 7, and 14 (n = 3).


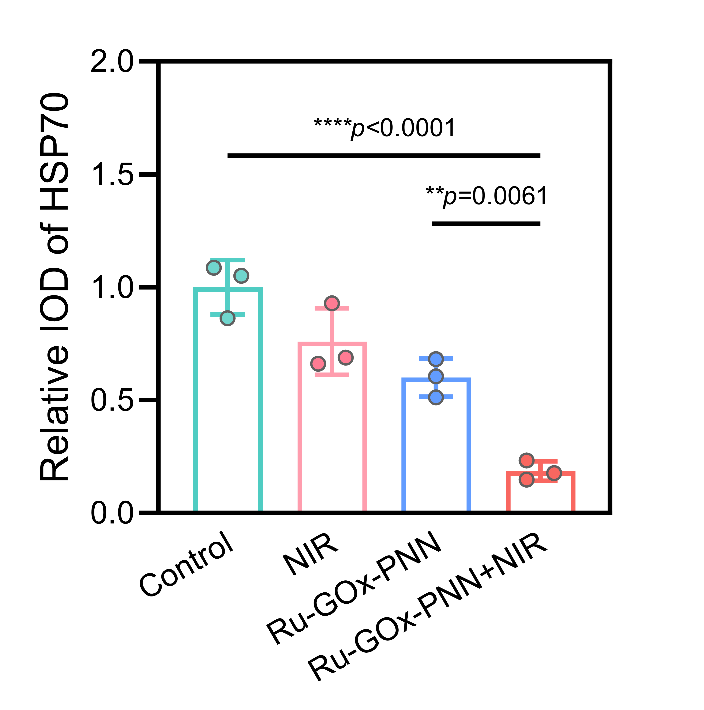
Figure S18. Quantitative analysis of HSP70 expression in tumor tissues from different treatment groups, determined by immunohistochemical staining (n = 3, one-way ANOVA with Tukey’s multiple comparisons test).


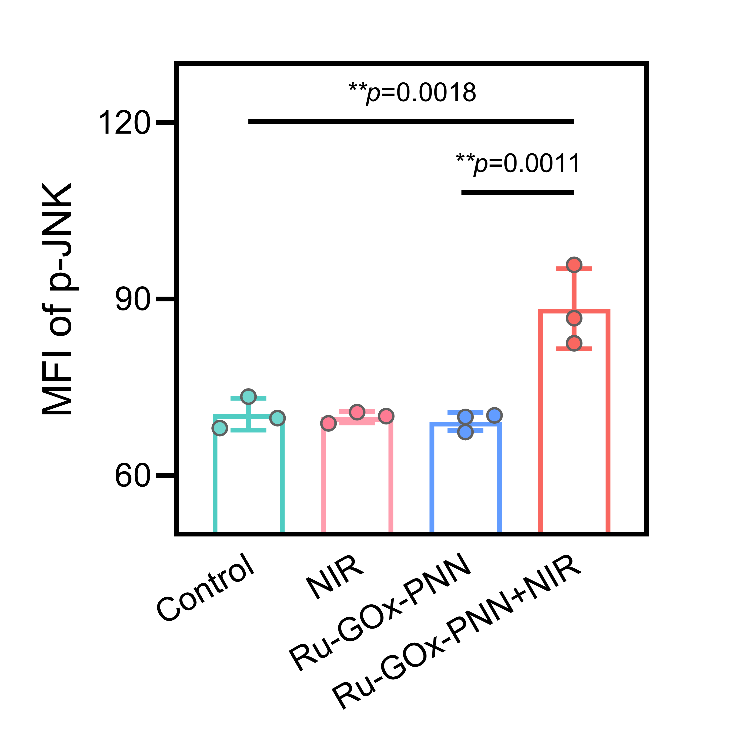


Figure S19. Quantification of p-JNK fluorescence signal in tumor tissue sections from each treatment group (n = 3, one-way ANOVA with Tukey’s multiple comparisons test).


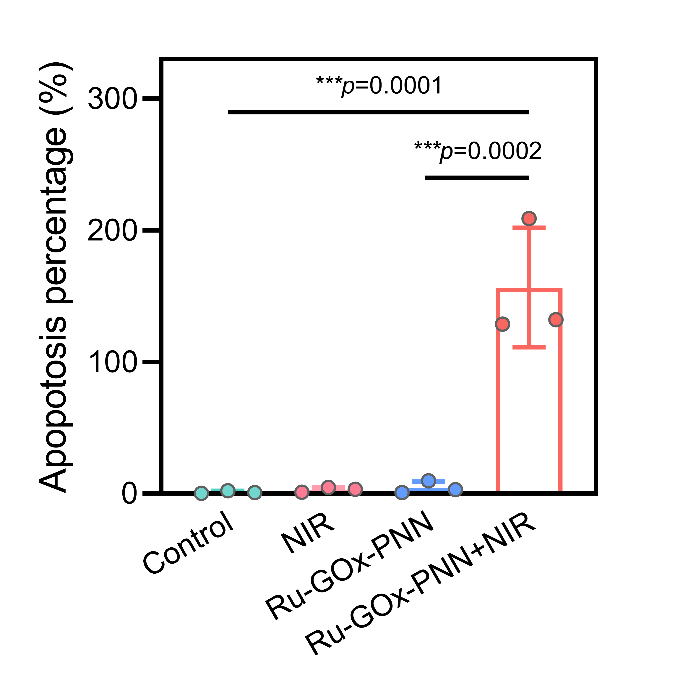


Figure S20. Quantification of TUNEL-positive apoptotic cells in tumor tissue sections from each treatment group (n = 3, one-way ANOVA with Tukey’s multiple comparisons test).


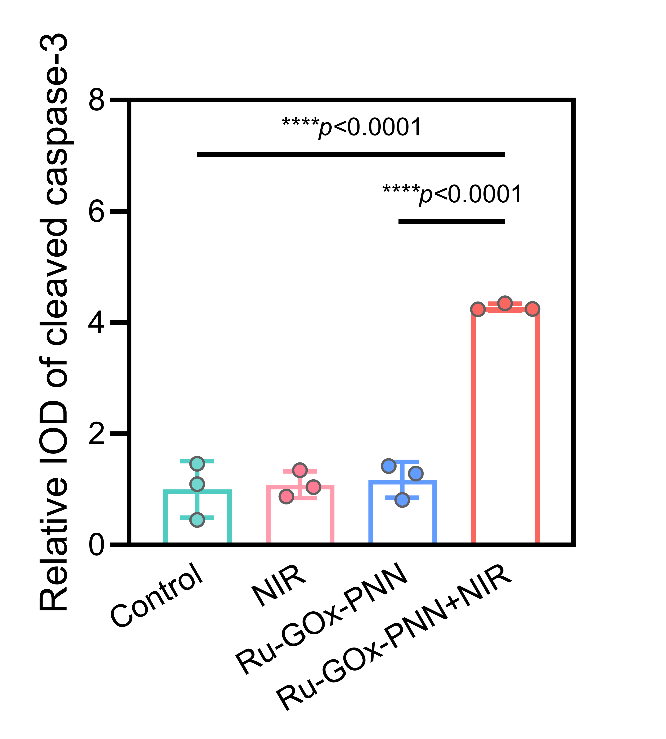
Figure S21. Quantitative analysis of cleaved caspase-3 expression in tumor tissues from different treatment groups, determined by immunohistochemical staining (n = 3, one-way ANOVA with Tukey’s multiple comparisons test).


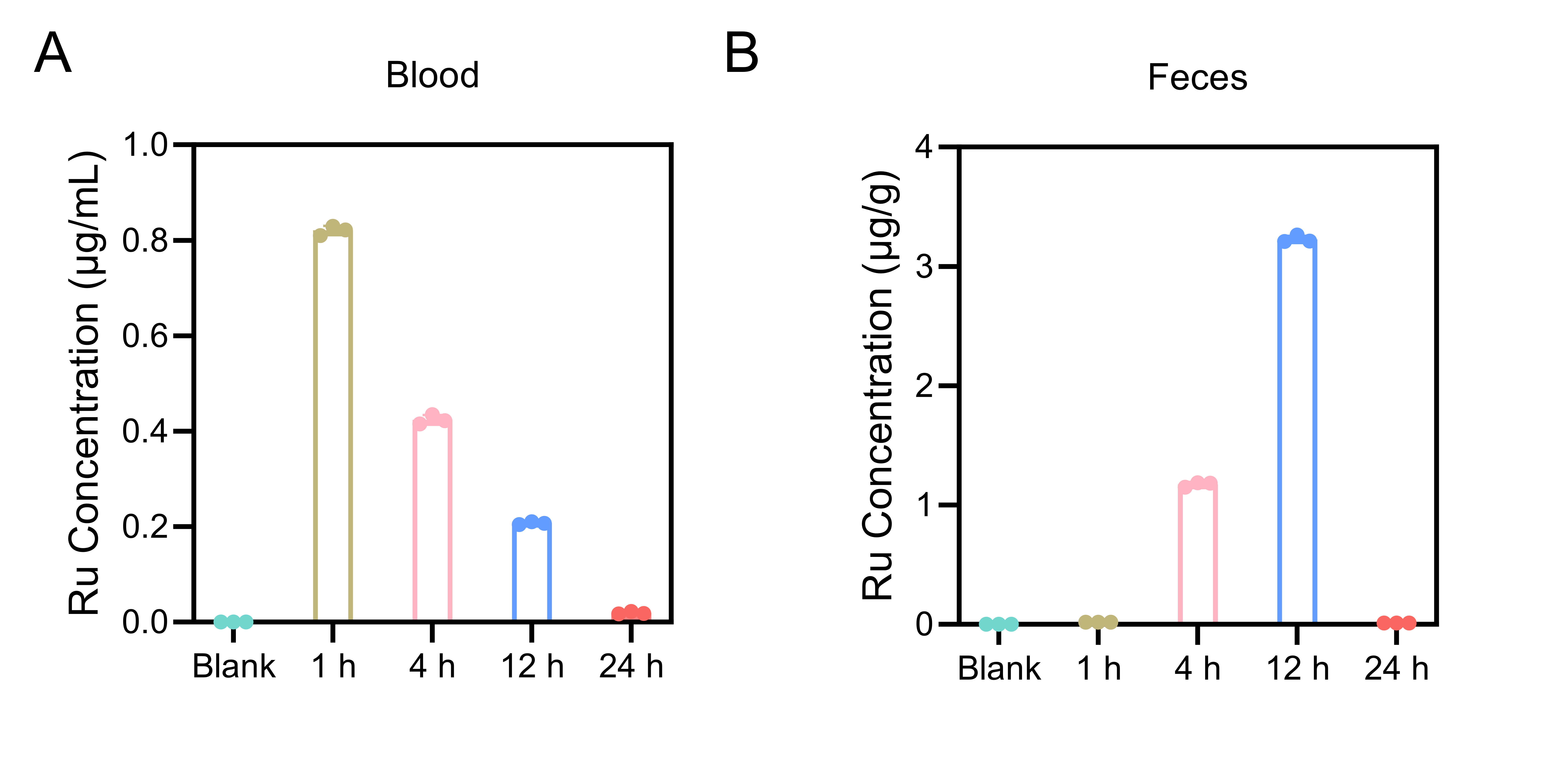
Figure S22. Analysis of Ru clearance *in vivo* by ICP-MS. A) Ru concentration in blood over time after administration of Ru-GOx-PNN. B) Ru content in feces collected at corresponding time point (n = 3).
