# Supplementary material for: Thermally Gated Dual‐Cascade Nanozyme for Enhanced Mild‐Temperature Photothermal Therapy
Source: Adv Sci (Weinh). 2025 Nov 7;13(18):e17528. doi: 10.1002/advs.202517528 (PMC13042585; doi:10.1002/advs.202517528)
Supplement: Supplementary file 2 — Supporting Information [file ADVS-13-e17528-s002.zip › STR - EC109.pdf]

Institute of Analysis and Testing,Beijing Academy of Science and Technology

（Beijing Center for Physical and Chemical Analysis）

Test Report

Project name	Cell line authentication
Client	Zhengzhou University
Date	2024.5.13-2024.6.6

Statement

1. The report is invalid without the official seal of the unit or the signature of the approver.
2. Any alterations, additions, deletions, or missing pages in the report are invalid. Multi-page reports must bear a continuous seal to be considered valid. Copies of this report are invalid without restamping the official seal.
3. This report is only responsible for the test data and results of the submitted samples. This report is for internal use only by the commissioning unit/person. Without the consent of this unit, the commissioning unit/person shall not use the test data and results of this report for publicity without authorization.
4. Any objections to the report must be raised by the commissioning unit/person within 15 days from the date of receipt. Failure to do so will result in non-acceptance of the objections.

1 Testing information:

Client: Zhengzhou University

Project name: Cell line authentication using STR profiling

Reference: Authentication of Human Cell Lines: Standardization of STR Profiling, ASN-0002-2011.

Testing sample:

Sample number	Sample name
240513-YFS-010	EC109

Date: 2024.6.5

2 Test methods

2.1 Isolation of Genomic DNA

Extraction of DNA using Chelex-100 was performed using 5% Chelex in sterile H_2O. 120 μL DNA extract was removed from the Chelex resin for further analysis.

2.2 STR profiling

STR profiling procedure was as follows: 21 STR loci (D3S1358, D5S818, D2S1338, TPOX, CSF1PO, Penta D, TH01, vWA, D7S820, D21S11, Penta E, D10S1248, D8S1179, D1S1656, D18S51, D12S391, D6S1043, D19S433, D16S539, D13S317, FGA and the amelogenin gender-determining marker were amplified using the SifaSTR 23 Plex ID system (Peoplespot, China) according to the manufacturer' s instructions. Electrophoretic analysis was carried out using 3130xl Genetic Analyzer (Applied Biosystems, USA). After electrophoresis, the data were analyzed by the Gene Mapper ID-X Software v3.0 (Applied Biosystems,

USA) to categorize peaks according to their size in relation to an internal standard allelic ladder.

2.3 Data Analysis

A minimum of eight core STR markers, D5S818, D13S317, D7S820, D16S539, vWA, TH01, TPOX, CSF1PO are necessary to uniquely identity human cells. Two cell lines with STR profiles identical at 7 or 8 loci of the 8 core STR markers are considered matching. STR data were analyzed using the ExPASy STR database (https://www.cellosaurus.org/str-search/). The submitted profile is an exact match for the following human cell line in the ExPASy STR database: EC109.

3 Results

3.1 The comparison results between the sample and reference data are shown in Table 1.

Table 1 The comparison results between the sample and reference data

	Test results for submitted sample	ExPASy Reference Database Profile
Loci	Query Profile: EC109	Database Profile: EC109
D3S1358	17	
D5S818	12	12
D2S1338	17, 20	
TPOX	8	8
CSF1PO	10	10
Penta D	9	
Indel		
Amel	X	
TH01	9	9
vWA	16, 17	16, 17
D7S820	8, 12	8, 12

D21S11	30	
Penta E	16	
D10S1248	13	
D8S1179	13, 14	
D1S1656	15, 16	
D18S51	12, 16	
D12S391	19, 20	
D6S1043	13	
D19S433	13	
D16S539	12	12
D13S317	11	11
FGA	25	

3.2 Online STR matching analysis with the ExPASy STR database showed that the test sample matched EC109 cell line.

Note: The test results are only applicable to the samples submitted for testing. This report is for scientific research, teaching, or internal quality control purposes only.

Tester	[illegible]	Date of issuance	2024.6.18
Approver	[illegible]	Date of issuance	2024.6.18
Unit Name (Official Seal)	**Institute of Analysis and Testing, Beijing Academy of Science and Technology（Beijing Center for Physical and Chemical Analysis）**		
Address	**4th Floor, Block B, Incubation Building, No.7 Fengxian Middle Road, Haidian District, Beijing 100094, P.R. China**		
