# Supplementary material for: Thermally Gated Dual‐Cascade Nanozyme for Enhanced Mild‐Temperature Photothermal Therapy
Source: Adv Sci (Weinh). 2025 Nov 7;13(18):e17528. doi: 10.1002/advs.202517528 (PMC13042585; doi:10.1002/advs.202517528)
Supplement: Supplementary file 2 — Supporting Information [file ADVS-13-e17528-s002.zip › Mycoplasma detection - EC109.pdf]

**Institute of Analysis and Testing, Beijing Academy of Science and Technology**

**（Beijing Center for Physical and Chemical Analysis）**

# Test Report

| | |
|---|---|
| Project name | Mycoplasma detection |
| Client | Zhengzhou University |
| Date | 2024.5.13-2024.5.23 |

# Statement

1. The report is invalid without the official seal of the unit or the signature of the approver.
2. Any alterations, additions, deletions, or missing pages in the report are invalid. Multi-page reports must bear a continuous seal to be considered valid. Copies of this report are invalid without restamping the official seal.
3. This report is only responsible for the test data and results of the submitted samples. This report is for internal use only by the commissioning unit/person. Without the consent of this unit, the commissioning unit/person shall not use the test data and results of this report for publicity without authorization.
4. Any objections to the report must be raised by the commissioning unit/person within 15 days from the date of receipt. Failure to do so will result in non-acceptance of the objections.

1 Testing information:

Client: Zhengzhou University

Project name: Mycoplasma detection

Testing sample:

| Sample number | Sample name |
|---|---|
| 240513-YFS-002 | EC109 |

Date: 2024.5.23

2 Test methods

2.1 Prepare the kit reagents and premix solution

2.1.1 Thaw all kit reagents completely.

2.1.2 Vortex briefly, then spin down the reagents.

2.1.3 Prepare the Premix Solution according to table 1.

Table 1. Prepare the Premix Solution

| Component for premix solution | Volume for one 40-μL reaction | Volume for six 40-μL reaction [1] |
|---|---|---|
| 4× MyqPCR Reaction Buffer | 10.0 μL | 66.0 μL |
| MyPrimer& Probe MIX | 1.0 μL | 6.6 μL |
| internal control (IC) | 1.0 μL | 6.6 μL |
| ROX | 0.4 μL | 2.64 μL |
| Nuclease free PCR-grade water | 7.6 μL | 50.16 μL |
| Total | 20 μL | 132.0 μL |

[1] Includes 10% excess to compensate for piptetting errors.

2.1.4 Mix the Premix Solution by gently pipetting up and down, then cap the tube.

2.2 Prepare the PCR reactions:

2.2.1 Dispense the following into each well to be used, gently pipetting at the bottom of the well

Table 2. Prepare the PCR reactions

| To prepare... | In each tube or well... |
|---|---|
| TS | Add 20 μL Premix Solution |

| | |
|---|---|
| | Add 20 μL unknown sample |
| NTC | Add 20 μL Premix Solution |
| | Add 20 μL DNA Dilution solution |
| PCS | Add 20 μL Premix Solution |
| | Add 20 μL positive control (PCS) |
| Total | 40 μL |

2.2.2 Mix each sample by gently pipetting up and down.

2.2.3 Seal the plate with MicroAmp Optical Adhesive Film.

2.2.4 Briefly centrifuge the reaction plate.

2.3 Set up and run the real-time PCR instrument

2.3.1 See the appropriate instrument user guide for detailed instructions to set up and run the real-time PCR instrument.

2.3.2 Set up the real-time PCR instrument using the following settings:

- Reaction volume: 40μL
- TaqMan probe reporter of Target 1: FAM dye, TaqMan probe quencher: None
- TaqMan probe reporter of Target 2: VIC dye, TaqMan probe quencher: None
- Thermal-cycling profile － See the table 3:

Table 3 Thermal-cycling profile

| Stage | Temperature | Time | Cycles | Data collection |
|---|---|---|---|---|
| 1 | 95°C | 5min | 1 | No |
| 2 | 95°C | 15sec | 45 | No |
| 3 | 62°C | 30sec | | FAM or VIC dye |

2.3.3 Load the reactions, run the thermal cycler program and collect real-time amplification data.

3 Data analysis

View the amplification plots for all reactions and check each sample for FAM and VIC dye signals. We use the default baseline and threshold values in the SDS analysis software. The results are listed in the table 4.

Table 4. The results of real-time PCR

| Sample Name | Target Name | Reporter | Quencher | $C_T$ |
|---|---|---|---|---|
| EC109 | Target 1 | FAM | NONE | Undetermined |
| EC109 | Target 1 | FAM | NONE | Undetermined |
| NTC | Target 1 | FAM | NONE | Undetermined |
| NTC | Target 1 | FAM | NONE | Undetermined |
| PCS | Target 1 | FAM | NONE | 27.30531 |
| PCS | Target 1 | FAM | NONE | 27.07055 |
| EC109 | Target 2 | VIC | NONE | 23.47042 |
| EC109 | Target 2 | VIC | NONE | 23.29285 |
| NTC | Target 2 | VIC | NONE | 24.27825 |
| NTC | Target 2 | VIC | NONE | 24.30766 |
| PCS | Target 2 | VIC | NONE | 24.0841 |
| PCS | Target 2 | VIC | NONE | 24.10176 |

## 4 Results

Determine whether the Target 1 and Target 2 can be detected for EC109. Target 1 PCR is not detected and Target 2 PCR is detected in EC109. It is shown that no mycoplasma is detected in EC109.

Note: The test results are only applicable to the samples submitted for testing. This report is for scientific research, teaching, or internal quality control purposes only.

| | | | |
|---|---|---|---|
| Tester | 張小莉 | Date of issuance | 2024. 6. 18 |
| Approver | [illegible] | Date of issuance | 2024. 6.18 |
| Unit Name (Official Seal) | **Institute of Analysis and Testing, Beijing Academy of Science and Technology （Beijing Center for Physical and Chemical Analysis）** | | |
| Address | **4th Floor, Block B, Incubation Building, No.7 Fengxian Middle Road, Haidian District, Beijing 100094, P.R. China** | | |
